# Global Antimicrobial Resistance Gene Study of *Helicobacter pylori*: Comparison of Detection Tools, ARG and Efflux Pump Gene Analysis, Worldwide Epidemiological Distribution, and Information Related to the Antimicrobial-Resistant Phenotype

**DOI:** 10.3390/antibiotics12071118

**Published:** 2023-06-28

**Authors:** Ricky Indra Alfaray, Batsaikhan Saruuljavkhlan, Kartika Afrida Fauzia, Roberto C. Torres, Kaisa Thorell, Selva Rosyta Dewi, Kirill A. Kryukov, Takashi Matsumoto, Junko Akada, Ratha-korn Vilaichone, Muhammad Miftahussurur, Yoshio Yamaoka

**Affiliations:** 1 Department of Environmental and Preventive Medicine, Faculty of Medicine, Oita University, Oita 879-5593, Japan; rickyindraalfaray@gmail.com (R.I.A.); saruuljavkhlan@yahoo.com (B.S.); kartikafauzia@oita-u.ac.jp (K.A.F.); m22d9104@oita-u.ac.jp (S.R.D.); tmatsumoto9@oita-u.ac.jp (T.M.); akadajk@oita-u.ac.jp (J.A.); 2 *Helicobacter pylori* and Microbiota Study Group, Institute of Tropical Disease, Universitas Airlangga, Surabaya 60286, Indonesia; 3 Department of Public Health and Preventive Medicine, Faculty of Medicine, Universitas Airlangga, Surabaya 60132, Indonesia; 4 The Center for Microbes, Development and Health, Key Laboratory of Molecular Virology and Immunology, Institute Pasteur of Shanghai, Chinese Academy of Sciences, Shanghai 200031, China; rtorres@ips.ac.cn; 5 Department of Chemistry and Molecular Biology, Faculty of Science, University of Gothenburg, 405 30 Gothenburg, Sweden; kaisa.thorell@gu.se; 6 Biological Networks Laboratory, Department of Informatics, National Institute of Genetics, Shizuoka 411-8540, Japan; kirill-kryukov@nig.ac.jp; 7 Gastroenterology Unit, Department of Medicine, Faculty of Medicine, Thammasat University Hospital, Khlong Nueng 12120, Pathumthani, Thailand; rkv@tu.ac.th; 8 Center of Excellence in Digestive Diseases, Thammasat University, Thailand Science Research and Innovation Fundamental Fund, Bualuang ASEAN Chair Professorship at Thammasat University, Khlong Nueng 12121, Pathumthani, Thailand; 9 Department of Medicine, Chulabhorn International College of Medicine (CICM), Thammasat University, Khlong Nueng 12121, Pathumthani, Thailand; 10 Division of Gastroentero-Hepatology, Department of Internal Medicine, Faculty of Medicine, Dr. Soetomo Teaching Hospital, Universitas Airlangga, Surabaya 60286, Indonesia; 11 The Research Center for GLOBAL and LOCAL Infectious Diseases (RCGLID), Oita University, Oita 870-1192, Japan; 12 Department of Medicine, Gastroenterology and Hepatology Section, Baylor College of Medicine, Houston, TX 77030, USA

**Keywords:** *Helicobacter pylori*, antimicrobial resistance (AMR), antimicrobial-resistant gene (ARG), genome analysis, global distribution, clinical implications

## Abstract

We conducted a global-scale study to identify *H. pylori* antimicrobial-resistant genes (ARG), address their global distribution, and understand their effect on the antimicrobial resistance (AMR) phenotypes of the clinical isolates. We identified ARG using several well-known tools against extensive bacterial ARG databases, then analyzed their correlation with clinical antibiogram data from dozens of patients across countries. This revealed that combining multiple tools and databases, followed by manual selection of ARG from the annotation results, produces more conclusive results than using a single tool or database alone. After curation, the results showed that *H. pylori* has 42 ARG against 11 different antibiotic classes (16 genes related to single antibiotic class resistance and 26 genes related to multidrug resistance). Further analysis revealed that *H. pylori* naturally harbors ARG in the core genome, called the ‘Set of ARG commonly found in the Core Genome of *H. pylori* (ARG-CORE)’, while ARG-ACC—the ARG in the accessory genome—are exclusive to particular strains. In addition, we detected 29 genes of potential efflux pump-related AMR that were mostly categorized as ARG-CORE. The ARG distribution appears to be almost similar either by geographical or *H. pylori* populations perspective; however, some ARG had a unique distribution since they tend to be found only in a particular region or population. Finally, we demonstrated that the presence of ARG may not directly correlate with the sensitive/resistance phenotype of clinical patient isolates but may influence the minimum inhibitory concentration phenotype.

## 1. Introduction

*Helicobacter pylori* is a stomach Gram-negative pathogen that infects around half of the global human population, affecting more than 4 billion people [1,2,3]. Persistent infection of this bacterium can lead to various clinical implications, including gastritis, gastric atrophy, gastric peptic ulcers, gastric intestinal metaplasia, mucosa-associated lymphoid tissue lymphoma, and gastric cancer [4]. As an effective vaccine for *H. pylori* is yet to be discovered, curative treatment using antimicrobial drugs remains crucial to prevent further transmission and resolve disease augmentation [4,5]. However, several issues have arisen, including bad practices in the use of antibiotics leading to the antimicrobial resistance (AMR) phenomenon, which has become a leading concern for *H. pylori* eradication in the last decade [6,7,8]. 

The molecular driving mechanisms of AMR in *H. pylori* are already well-studied [5]. However, most studies merely focus on the mutation of antibiotic target alteration genes, despite the ability for the acquisition or loss of antimicrobial-resistant genes (ARG) by processes such as horizontal gene transfer (HGT) [9]. One reason is that mutation is believed to be the primary mechanism of AMR development in clinical conditions [5]. Nevertheless, this has resulted in less study of the current worldwide epidemiology of ARG in *H. pylori*. Thus, a global-scale study needs to be undertaken to understand the current landscape of *H. pylori* ARG epidemiology.

Several challenges need to be addressed, beginning with the preferred tools to better detect ARG in the *H. pylori* genome, the global *H. pylori* ARG distribution, and the impact of the presence of ARG on the clinical AMR phenotype. Hence, we attempted to address these challenges using a global *H. pylori* genome collection covering 68 countries from 5 different continents. We focused on ARG against drugs or other potential antimicrobial substances. In this study, we defined ARG as any potential acquired antimicrobial resistance-related gene listed in public ARG databases that include AMR-associated genes tested in many bacterial species (including members of the family *Helicobacteraceae*), but excluding most of well-known genes covering AMR through mutations, such as the mutations in *pbp1A* (ß-lactam-resistant), *gyrA* (fluoroquinolones-resistant), *23S rRNA* (macrolides-resistant), *16S rRNA* (tetracycline-resistant), and *rpoB* (ryfamycins-resistant), which mainly belong to the antibiotic target alteration gene, also known as drug target-mediated resistance or modification of the drug target [5]. Based on these criteria, we highlighted the global epidemiology of ARG in *H. pylori* and found that most are yet to be experimentally proven. In addition, we also analyzed the potential efflux pumps (EPs)-related AMR. These results will lead to many opportunities for future *H. pylori* ARG studies. Finally, we also attempted to identify new ARG candidates in *H. pylori* using antibiogram data retrieved from multinational clinical patient isolate strains.

## 2. Results

### 2.1. ARG Detection Tools and Comparison of Results

The ARG detection tools and methods varied between *H. pylori* genome studies. While some studies simply identified ARG using tools working with the BLAST-matches-based method for the nucleotide sequence [10,11], other studies have identified ARG using tools that work mainly with amino acid sequence database homology and single-nucleotide polymorphism (SNP) models [12,13]. Each tool was also used with different parameters (e.g., identity, coverage, e-value, and bit score), resulting in variable results between studies. These tools also rely on databases that are mostly not curated for *H. pylori*. To the best of our knowledge, there is no guideline for ARG detection purposes in the *H. pylori* study field. Thus, we tried to compare tools with different methods that can be used for ARG detection for *H. pylori*. These tools work with the BLAST-matches-based method, the homology and SNP model-based methods, the Hidden Markov model (HMM) screening method, or a combination of these (Table 1).

Initially, we performed ARG detection using the ABRICATE tool against several well-known ARG databases that use the BLAST-matches-based method [14]. Databases such as ARG-ANNOT [15], CARD [16,17], MEGARes [18], and ResFinder [19] are commonly used to identify ARG in the prokaryotic genome [20]. Therefore, we used these databases to obtain optimum results. In addition, we compared three different parameters for minimum identity and coverage: cov = 50 id = 50, cov = 70 id = 70, and cov = 90 id = 90. The results of these parameter sets were nearly the same, with the only slight difference being because of the coverage, as all results had the same identity of more than 90% (Figure S1). Therefore, using this tool’s parameter set of cov = 90 id = 90 for ARG detection could be a wise option to obtain more accurate results. 

**Figure 1:**
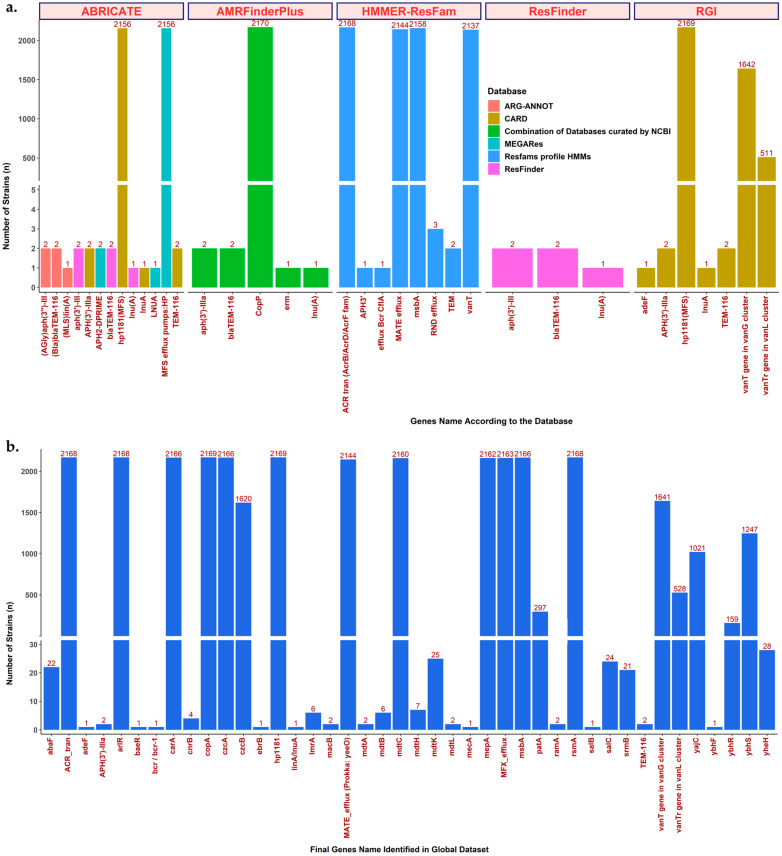
ARG detection by several tools, methods, and databases. (**a**) The ARG detection results are based on well-known ARG detection tools, showing that each tool could yield different results since they have different detection methods or databases. (**b**) Final ARG detection results in the global *H. pylori* dataset. A total of 42 ARG were included after collecting the results from all tools and databases, then performing careful curation with Prokka annotation to delete duplication, confirm the presence of the gene, and find undetected ARG.

Using the parameter settings of minimum coverage and identity set to 90, we found 5 strains containing 5 genes from 3 different ARG using ARG-ANNOT, 2161 strains containing 2166 genes from 4 different ARG using CARD, 2159 strains containing 2162 genes from 3 different ARG using MEGARes, and 5 strains containing 5 genes from 3 different ARG using ResFinder (Figure 1a and Figure S1). The genes found by ARG-ANNOT were similar to those from ResFinder, and those found by CARD were the same as MEGARes, except for *TEM*. The CARD and MEGARes results included the results from ARG-ANNOT and ResFinder. Thus, the BLAST-matches-based method, using either the CARD or MEGARes database, yielded the best results for detecting ARG in *H. pylori* compared to other current databases. 

Although the ARG names between CARD and MEGARes were slightly different, they referred to the same genes. For instance, *hp1181* in CARD and *HP* in MEGARes both refer to the same Major Facilitator Superfamily (MFS). We confirmed this finding by aligning the gene sequences from both databases and the representative sequence from the *H. pylori* strain dataset (Figure S2a,b). Intriguingly, this gene was found in 99.35% (2156/2170) of the total strains. Each strain possessed one copy of this ARG, except for two strains (strain MS203 had three copies, and UM067 had two copies) (Spreadsheet S1). 

After ABRICATE, we performed ARG detection using ResFinder [19]. This tool was mainly employed using the BLAST-matches-based method [19]. ResFinder and ABRICATE differ in terms of their databases: the ResFinder database in ABRICATE only contains ARG, while the ResFinder tool database is more varied, especially because it also contains disinfectant resistance genes and chromosomal mutations [19]. We used the ResFinder tool against the acquired and disinfectant resistance gene databases, using the default parameters, with the minimum coverage and identity set as 90%. The result for the acquired resistance genes was the same as ABRICATE using the ResFinder database (Figure 1a, Spreadsheet S2). No anti-disinfectant genes were detected.

Next, we performed ARG detection using the Resistance Gene Identifier (RGI) [17]. This tool works using the homology and SNP model-based methods [17]. In brief, RGI will algorithmically predict ARG and mutations from genomes using a mix of Prodigal open reading frame prediction [21], BLAST [22] or DIAMOND [23] sequence alignment, and curated resistance mutations included in the AMR detection model [17]. We used the default parameters and included the results based on the ‘strict’ and ‘perfect’ criteria only, except for *hp1181*, which was mainly detected as ‘loose’. However, after hand curation, *hp1181* appeared in 99.95% (2169/2170) of the total strains. This result is accurate according to the previous version of the RGI (v5.2.1), where *hp1181* was included in the ‘strict’ group. Nevertheless, in any version of RGI, the default database is the CARD database [17]. Using RGI v6.0.1 and the CARD database v3.2.5, 2022, we identified 4328 genes from seven different ARG (Figure 1a, Spreadsheet S3). The difference to the ABRICATE results was that RGI could detect *vanT,* including its variant *vanTr*. RGI could also detect more strains containing ARG and more *hp1181* genes compared with ABRICATE (i.e., UM067 with two genes, N6 with two genes, MS203 with three genes, ASHA009 with two genes, and BGD53 with two genes). RGI could detect more ARG even if the genes were defined as a hypothetical protein by the Prokka annotation tool (Figure S3).

The next ARG detection tool was AMRFinderPlus, which combines BLAST-matches and the HMM screening method, with some improvements in the algorithm [24]. This tool is newly developed by the National Center for Biotechnology Information (NCBI) and combines nucleotide, protein, and HMM databases. We used the default parameters and added a ‘--plus’ flag to run AMRFinderPlus. Based on the recommendations of the software, we used the FASTA nucleotide assembly sequence (.fasta/.fna), protein sequence (.faa), and general feature format (.gff) to obtain the most accurate results [24]. We found 2176 genes from 5 different ARG based on AMRFinderPlus (Figure 1a, Spreadsheet S4). The difference between this tool and the other tools is that AMRFinderPlus detected *copA*, a gene encoding copper-binding metallochaperone CopP, in all strains.

Finally, the last ARG detection tool was HMMER, against the HMM ResFam Profile Database [25,26]. This tool specifically uses the HMM screening method. We used the parameter setting of e-value ≤ 1 × 10^−10^. As for the bit score, this score setting should be relative to every analysis, and we should understand the good score for the ideal query to obtain the best results while using the HMM ResFam Profile Database. Therefore, we compared results with different bit score values (≥100, ≥200, ≥250, ≥300, ≥400, and ≥500) and then evaluated the resul ts by hand curation against Prokka annotation results for several randomly selected representative strains that, relatively, had a higher number of ARG detected (Spreadsheet S5). We excluded the results if they contained antibiotic target alterations or unclear information (e.g., no clear information about the antibiotic class affected by the presence of the gene). The results implied that setting the score at ≥100 and ≥200 yielded many false-positive results (genes were detected as ARG but were actually various functional genes). In addition, many genes were detected with the same ARG name as if they were a copy number or variant, but each gene was actually different. The score ≥ 250 yielded a more accurate result, with particular caution for *ACR_tran* and *msbA,* as these genes often appear to have multiple copy numbers or variants when only one is correct and the other is actually a different gene. The score ≥ 500 yielded a more accurate result; however, many ARG could be left undetected. Thus, a bit score ≥ 250 should be used for *H. pylori*; however, the result should be treated carefully, for example, with further hand curation against genome annotation tool results (e.g., Prokka, RAST, PGAP, or D-FAST). Many inconsistencies of the annotation results could be found, with several genes annotated with different names compared to the Prokka annotation (e.g., MATE_efflux should be *yeeO* by Prokka). Therefore, careful hand curation, including comparison of the ResFam updated metadata and Prokka results (e.g., the gene’s name, location, similarity, and other information), is recommended. Finally, a total of 15,056 genes covering 7 ARG were found after hand curation, excluding inconsistent results and modification of the drug target (mutation-related ARG) (Spreadsheet S5). 

An interesting finding was that one of the most predominant ARG was *hp1181*, or the MFS EPs family (found in 99.86%; 2167/2170 of the total strains based on HMMER against the HMM ResFam Profile Database). This gene was found by HMMER, RGI, and ABRICATE using the CARD or MEGARes databases, both of which contained a few *H. pylori* ARG. According to the data in CARD, this EPs is related to fluoroquinolone, nitroimidazole, and tetracycline antibiotic resistance [16,27]. The expression levels of these genes correlate with AMR development, but the protein structural analysis and functional mechanism in *H. pylori* remain unclear [27]. Therefore, we tried to predict the structure and function by looking for the closest protein reported in the Protein Data Bank (PDB) database. Investigation of the similarity of this protein against protein databases using Phyre2 showed that this MFS EPs structure is similar to the ‘drug efflux protein—MFS transporter’ reported in *E. coli* [28,29,30]. By Phyre2, as much as 99% of the amino acid template from the *H. pylori* MFS could be modeled with 100.0% confidence and 99% coverage against the *E. coli* MFS [29,30]. Next, we used a structure comparison tool via the SWISS-MODEL and MASS to confirm the similarity between *H. pylori* and *E. coli* MFS [31,32]. We built a new protein model of the *H. pylori* MFS using AlphaFold2 to yield a more accurate protein structure [33]. Overall, the results suggested that these structures have similarity (average root mean square deviation (RMSD) = 0.84) to *E. coli* (Figure S4); thus, it might have the same function in *H. pylori* as the *E. coli* MFS, that is, to help the bacteria becoming resistant to particular antibiotics. 

**Table 1:** Tools, methods, and databases that are often used for *H. pylori* ARG detection.

Tool	Method	Database	Provide Option for Mutation Detection	*H. pylori* ARG is Included	Database Version, Year Updated	Total Number of Genes Found in 2170 Strains	Total Number of Strains with ARG	Total ARG Name Found	Total ARG Class Found	Parameter Used
ABRICATE v1.0.1 [14]	BLAST-matches	ARG-ANNOT [15]	No	Not yet	v5, 2019	5	5	3	3	Minimum coverage and identity of 90
		CARD [16,17]	No	Yes, but limited	March 2020 update	2166	2161	4	4	
		MEGARes [18]	No	Not yet	v2.0, 2020	2162	2159	3	3	
		ResFinder [19]	No	Yes	2019	5	5	3	3	
ResFinder v4.2.3 [19]	The aligners KMA and BLAST-matches	ResFinder and DisinFinder [19]	Yes	Yes, but limited	2022	5	5	3	3	Minimum coverage and identity of 90
The Resistance Gene Identifier (RGI) v6.0.1 [17]	Homology and SNP models	CARD [16,17,34]	Yes	Yes, but limited	v3.2.5, 2022	4328	2170	7	4	Default parameter and filter to obtain the ‘strict’ and ‘perfect’ results only
AMRFinderPlus v3.11.2 [24]	Combination of BLAST-matches, HMM screening, and other improvement methods	Combination of the following: Pathogen Detecton Reference Gene Catalog (https://www.ncbi.nlm.nih.gov/pathogens/refgene/#, accessed on 22 July 2021) Pathogen Detecton Reference HMM Catalog (https://www.ncbi.nlm.nih.gov/pathogens/hmm/, accessed on 22 July 2021) Bacterial Antimicrobial Resistance Reference Gene Database (https://www.ncbi.nlm.nih.gov/bioproject/PRJNA313047, accessed on 22 July 2021) NCBIfam-AMRFinder (https://ftp.ncbi.nlm.nih.gov/hmm/NCBIfam-AMRFinder/latest/, accessed on 22 July 2021)	Yes	Not yet	v2022-12-19.1, 2022	2176	2170	5	4	Default parameter, add’—plus’ flag, and three files typed as input (.fna, .faa, and .gff)
HMMER v3.3.2 [25]	HMM screening	HMM ResFam Profile [26], which was trained using ARG protein sequence from CARD, the Lactamase Engineering Database (LacED), and Jacoby and Bush’s collection of curated beta-lactamase proteins.	Possible	Not yet	v1.2.2, 2018	15,056	2170	7	10	e-value ≤ 1 × 10^−10^ and bit score ≥ 250 after hand curation

### 2.2. Pros and Cons of ARG Detection Tools

If we simply wished to perform an analysis targeting only complete genes and expecting only genes with high-similarity nucleotide sequences as the result, a convenient tool using BLAST-matches-based methods, such as ABRICATE, is a good choice, as the results are expected to only represent the complete genes. However, while the results are most likely accurate, these results will be limited because it will not detect as many ARG as other tool results. Another tool with the same BLAST-matches-based method, such as ResFam or ResFinder, might not provide the optimum results for ARG detection in *H. pylori*. The ResFam database does not contain specific *H. pylori* ARG compared with the CARD or MEGARes databases, while ResFinder is not as comprehensive as the CARD or MEGARes databases. The advantage of ResFinder is that it can also detect mutations related to AMR, as this tool applies KMA (k-mer alignment).

Next, for the RGI, our observations suggested that there are three advantages of RGI compared with ABRICATE and ResFinder. The first is that detection becomes more accurate and easier when we use assembly sequence as the input, because when we submit a nucleotide sequence, RGI first uses Prodigal to predict entire open reading frames (ORFs) and then evaluates the predicted protein sequences. This includes a subsequent correction by RGI if Prodigal misses the correct start codon, to anticipate entire AMR genes. Thus, it may detect more ARG even though the gene sequence might be partially different from the input and is often described as a hypothetical protein by the annotation tool (Figure S3). The second advantage is that it can also detect some mutations of antibiotic target alteration genes in *H. pylori* compared to other tools. This study found 611 *23S rRNA* genes with 3 well-known AMR mutations. Since these genes were not analogous to the focus of our study, we omitted these genes from the analysis results. The third advantage is that RGI could detect partial genes (e.g., we detected two MFS in BGD53, one complete while the other was partial). The consequence of this, that can become a limitation of this method, is that careful parameter measurement is required if we want to filter out these partial genes from our analysis. 

Finally, for the HMM screening method, in this study, this method could detect more ARG variants compared with ABRICATE, ResFam, and RGI. The HMMER algorithms can predict genes that cannot be easily detected only using nucleotide BLAST-matches-based (mass screening) methods [25]. This method will detect genes with relatively high nucleotide sequence variation. For example, the outer membrane efflux protein (OEP) family often has poor sequence conservation in several species, including *H. pylori* [35]. The HMM ResFam profile was constructed using ResFam and other databases, including TIGRFam [26]. The developer compared HMMs from ResFam with pairwise sequence alignment (BLAST) against the CARD and ARDB databases to evaluate their ability to predict AR function and found that ResFam detected more genes compared to the others [26]. However, for *H. pylori* genome analysis, hand curation is strongly recommended before drawing conclusions from the results because many false positives can be detected. Although the curation step is time-consuming, it will yield more accurate and conclusive results.

### 2.3. Curation of ARG Detection Results Is Necessary

Detection of ARG should be followed by careful curation, as the results from the detection tools can vary depending on their built-in methods and databases. For example, this curation can be performed by matching the identified ARG results with the whole-genome annotation results. After combining the genes identified by all tools, including ‘loose’ hits by RGI, we performed curation, then confirmed whether these genes existed within the Prokka results. Next, hypothetical proteins from the Prokka results were assigned as ARG if they were identified as resistance genes by RGI in the ‘strict’ and ‘perfect’ parameters. This curation step provides high confidence to the ARG detection results. A total of 30 ARG were included after this curation step. 

In addition, we realized that Prokka could possibly find more ARG that sometimes could not be detected by ARG detection tools. Indeed, when we extracted genes that contained any of the following keywords: ‘resistant’, ‘resistancy’, ‘resistance’, ‘drug’, ‘antibiotic’, and ‘antimicrobial’, we newly found 12 ARG that were not yet identified by any of the ARG detection tools. Finally, a total of 42 ARG were used for the subsequent analyses in this study (Figure 1b, Spreadsheet S6). 

### 2.4. Summary of Global ARG Detection Results from All Tools and Databases

**Figure 2:**
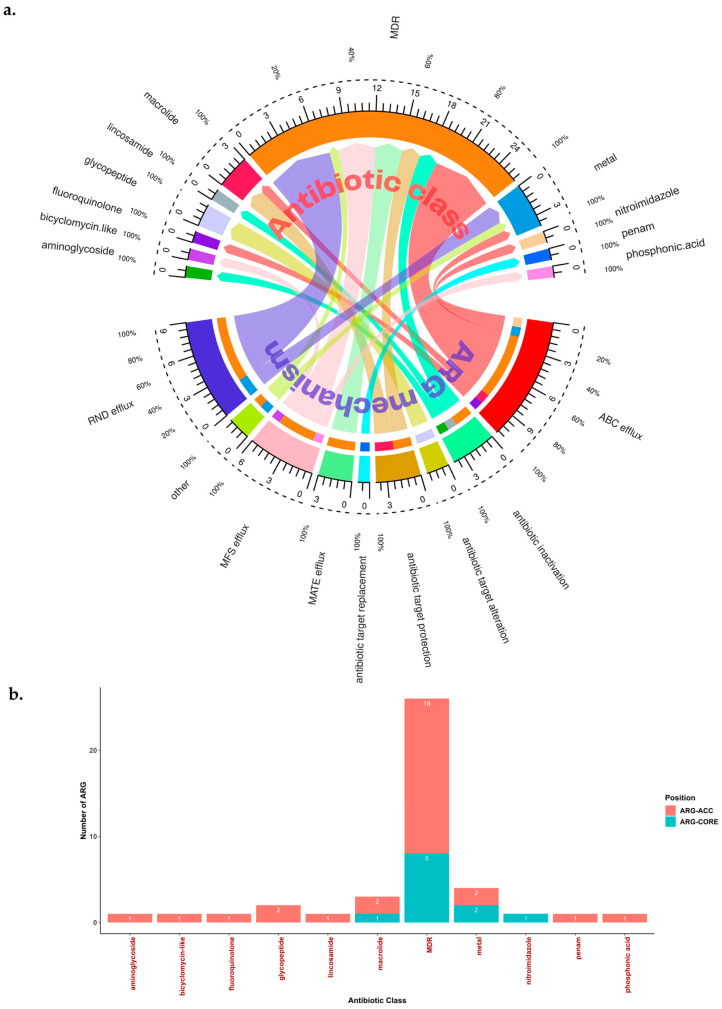
Antibiotic class, mechanism, and position of ARG identified within the *H. pylori* genome. (**a**) Schematic link between the ARG antibiotic class and the ARG resistance mechanism. Most of the MDR ARG were related to transporters or EPs. The inner and outer scales represent the number and proportion of ARG inside each antibiotic class. (**b**) Position of ARG within the *H. pylori* genome. The number of ARG represents the gene name that could be found within the antibiotic class. ARG-CORE: Set of ARG commonly found in the Core Genome of *H. pylori*. ARG-ACC: Set of ARG commonly found in the Accessory Genome of *H. pylori*. The core genome was defined if the ARG was found in ≥95% of the total global *H. pylori* dataset (*n* = 2170 strains).

Of the 42 ARG, 16 were related to single antibiotic class resistance (from 9 different antibiotic classes), while the other 26 were related to multidrug resistance (MDR) (Table 2). These 42 ARG have several resistant mechanisms, with the most common mechanism being correlated with EPs (69.05%; 29/42) [26] (Figure 2a). We found 4 major families of EPs in *H. pylori* ARG: Resistance Nodulation Cell Division (RND) efflux (31.03%; 9/29), ATP Binding Cassette (ABC) efflux (37.93%; 11/29), Major Facilitator Superfamily (MFS) efflux (20.69%; 6/29), and Multidrug and Toxin Extrusion (MATE) efflux (10.34%; 3/29). These EPs were related to single drug class resistance (27.59%; 8/29) or MDR (72.41; 21/29) (Figure 2a). 

Further in-depth genome observations revealed that 12 of the ARG could be found in the core genome (with the cut-off value of ≥95% of the total strains), while the other 30 could be found in the accessory genomes (Figure 2b). We called this ARG core genome the ‘Set of ARG commonly found in the Core Genome of *H. pylori*’ (ARG-CORE) (28.57%; 12/42), while ARG in the accessory genome were termed the ‘Set of ARG commonly found in the Accessory Genome of *H. pylori*’ (ARG-ACC) (71.43%; 30/42). Genes related to MDR were the most commonly found ARG in both ARG-CORE and ARG-ACC (66.67%; 8/12, and 60.00%; 18/30, respectively). Interestingly, genes such as *msbA* and *carA* were included in the ARG-CORE group, which aligns with the high prevalence of metronidazole and clarithromycin resistance in the current global *H. pylori* AMR status [7]. Other genes such as *vanT* and *vanTr* can be found in most of the strains (Table 2). Although these genes had a resistant mechanism as the antibiotic target alteration, we still included them in our analysis because the presence of *vanT* or *vanTr* may lead to a different phenotype which has not been thoroughly studied yet in *H. pylori*, thus, leading to an interesting opportunity for future study. This gene can be considered as ARG-CORE if *vanT* and *vanTr* together are considered as a same gene. However, we separated this gene following the tool and database identification results. Based on the identification results, *vanT* and *vanTr* will usually never be found in a single strain. Among other reasons, *vanT* can partially answer why *H. pylori* is naturally resistant to glycopeptides (e.g., vancomycin) [36].

### 2.5. Potential Efflux Pumps (EP)-Related AMR and MDR in H. pylori

Global ARG detection results showed several ARG belonging to EPs. Thus, other EPs that may correlate with AMR in *H. pylori* might be left undetected. Previous studies in *H. pylori* showed that several EPs could be related to AMR and MDR phenotypes in *H. pylori* [27,37,38,39,40,41,42,43,44,45,46,47]. A few of these EP genes were already identified in the ARG detection results (e.g., HP1181 in Figure 1b), while the others remain unidentified. This is because these databases (Table 1) may not contain all the EPs related to AMR in *H. pylori*. Therefore, we tried to construct a special query database containing EP sequences that were previously reported to correlate with AMR in *H. pylori*. A total of 28 EPs related to AMR or MDR based on previous reports were included, including EP genes that were identified by the ARG detection tools (Table S1) [27,37,38,39,40,41,42,43,44,45,46,47]. We added one more EP gene which detected because the name containing the ‘keywords’ (i.e., HP1120), therefore, the total of EPs-related AMR becomes 29 genes. Next, we tried to detect these EP genes using the BLASTN method against 2170 WGS. Initially, we tried to compare different parameters of identity and coverage to obtain the optimum results. Parameter identity and coverage of 50, 70, and 80 showed comparable results in all the EP genes, while identity and coverage of 90 showed a different value for HP1250 and HP1561. The total number of HP1250 should be almost similar to HP1251 and HP1252 since they are a set of ABC EPs (Figure S5). Notably, depending on the objective, higher identity and coverage should yield fewer false-positive results for simple presence/absence analysis using BLASTN. Thus, a parameter identity and coverage of 80 might be the optimum settings to detect EP genes in *H. pylori*, as shown at least in this study setting. The EP detection results showed that almost all EP genes were ARG-CORE, except HP1206 and HP0600 (Table 2). Generally, each strain will have one of each EP gene. 

**Table 2:** Summary of ARG detected by tools after curation and EPs related to AMR.

Gene Name (According to the Databases)	ARG-CORE (Detected in ≥95% of Total Strains) or ARG-ACC	Antibiotic Target	Resistance Mechanism	Additional Information (Including Gene Description by Prokka or Protein Homologous Name or Another Alternative Name ^a^)	AMR Gene Family (by CARD)	Prevalence in Total Genome Dataset (*n* = 2170)
**ARG detected by tools after curation**						
*abaF*	ARG-ACC	phosphonic acid antiobiotic	MFS efflux	Major Facilitator Superfamily (MFS) antibiotic efflux pump; fosfomycin resistance protein AbaF	Major Facilitator Superfamily (MFS) antibiotic efflux pump	1.01% (22/2170)
*adeF*	ARG-ACC	MDR (e.g., tetracycline, fluoroquinolone)	RND efflux	-	resistance-nodulation-cell division (RND) antibiotic efflux pump	0.05% (1/2170)
*APH*(3)-IIIa	ARG-ACC	aminoglycoside	antibiotic inactivation	-	APH(3′)	0.09% (2/2170)
*arlR*	ARG-CORE	MDR (e.g., fluoroquinolone, disinfecting agents, and antiseptics)	MFS efflux	Response regulator ArlR	Major Facilitator Superfamily (MFS) antibiotic efflux pump	99.91% (2168/2170)
*baeR*	ARG-ACC	MDR (e.g., aminocoumarin antibiotic, aminoglycoside antibiotic)	RND efflux	Transcriptional regulatory protein BaeR	resistance-nodulation-cell division (RND) antibiotic efflux pump	0.05% (1/2170)
*bcr-1*	ARG-ACC	Bicyclomycin-like antibiotic (it is also possible as MDR)	MFS efflux	Bicyclomycin resistance protein	Major Facilitator Superfamily (MFS) antibiotic efflux pump	0.05% (1/2170)
*TEM*-*116*	ARG-ACC	MDR	antibiotic inactivation	-	TEM beta-lactamase	0.09% (2/2170)
*carA*	ARG-CORE	macrolide	antibiotic target protection	-	Miscellaneous ABC-F subfamily ATP-binding cassette ribosomal protection proteins	99.82% (2166/2170)
*cnrB*	ARG-ACC	metal	other	Nickel and cobalt resistance protein CnrB	4-hydroxy-tetrahydrodipicolinate synthase	0.18% (4/2170)
*copA*	ARG-CORE	metal	ABC efflux	Copper-exporting P-type ATPase	-	99.95% (2169/2170)
*czcA*	ARG-CORE	metal	RND efflux	Cobalt-zinc-cadmium resistance protein CzcA	-	99.82% (2166/2170)
*czcB*	ARG-ACC	metal	RND efflux	Cobalt-zinc-cadmium resistance protein CzcB	-	74.65% (1620/2170)
*ebrB*	ARG-ACC	MDR (e.g., carbapenem, cephalosporin, penam)	antibiotic inactivation	-	Multidrug resistance protein EbrB	0.05% (1/2170)
*hp1181*	ARG-CORE	MDR (e.g., tetracycline, nitroimidazole, fluoroquinolone)	MFS efflux	in Prokka, can be detected as *yfcJ;* putative MFS-type transporter YfcJ	Major Facilitator Superfamily (MFS) antibiotic efflux pump	99.95% (2169/2170)
*lmrA*	ARG-ACC	MDR (e.g., lincosamide antibiotic)	ABC efflux	Multidrug resistance ABC transporter ATP-binding and permease protein	-	0.28% (6/2170)
*lnuA*	ARG-ACC	lincosamide	antibiotic inactivation	*linA*	lincosamide nucleotidyltransferase (LNU)	0.05% (1/2170)
*macB*	ARG-ACC	macrolide	ABC efflux	*pvdT*	ATP-binding cassette (ABC) antibiotic efflux pump	0.09% (2/2170)
*mdtA*	ARG-ACC	MDR (e.g., aminocoumarin)	RND efflux	*yegM*; Multidrug resistance protein MdtA	resistance-nodulation-cell division (RND) antibiotic efflux pump	0.09% (2/2170)
*mdtB*	ARG-ACC	MDR (e.g., aminocoumarin)	RND efflux	*yegN*; Multidrug resistance protein MdtB	resistance-nodulation-cell division (RND) antibiotic efflux pump	0.28% (6/2170)
*mdtC*	ARG-CORE	MDR (e.g., aminocoumarin)	RND efflux	*yegO*; Multidrug resistance protein MdtC	resistance-nodulation-cell division (RND) antibiotic efflux pump	99.54% (2160/2170)
*mdtH*	ARG-ACC	MDR (e.g., fluoroquinolone antibiotic)	MFS efflux	*yceL*; Multidrug resistance protein MdtH	resistance-nodulation-cell division (RND) antibiotic efflux pump	0.32% (7/2170)
*mdtK*	ARG-ACC	MDR (e.g., aminocoumarin)	MATE efflux	*norE*; *norM*; *ydhE*; Multidrug resistance protein MdtK	resistance-nodulation-cell division (RND) antibiotic efflux pump	1.15% (25/2170)
*mdtL*	ARG-ACC	MDR (e.g., aminocoumarin)	MFS efflux	*yidY*; Multidrug resistance protein MdtL	resistance-nodulation-cell division (RND) antibiotic efflux pump	0.09% (2/2170)
*mecA*	ARG-ACC	penam	antibiotic target replacement	Adapter protein MecA	Methicillin-resistant PBP2	0.05% (1/2170)
*mepA*	ARG-CORE	MDR (e.g., tetracycline, glycylcycline)	MATE efflux	Multidrug export protein MepA	multidrug and toxic compound extrusion (MATE) transporter	99.63% (2162/2170)
*MFS*_*efflux*	ARG-CORE	MDR	ABC efflux	HP1120 (COG1131); Multidrug efflux system ATP-binding protein	-	99.68% (2163/2170)
*msbA*	ARG-CORE	nitroimidazole	ABC efflux	Lipid A export ATP-binding/permease protein MsbA	ATP-binding cassette (ABC) antibiotic efflux pump	99.82% (2166/2170)
*patA*	ARG-ACC	fluoroquinolone	ABC efflux	Peptidoglycan O-acetyltransferase	ATP-binding cassette (ABC) antibiotic efflux pump	13.69% (297/2170)
*ramA*	ARG-ACC	MDR (e.g., tetracycline, rifamycin, phenicol, carbapenem, penem, penam, cephalosporin, cephamycin, glycylcycline, disinfecting agents and antiseptics, monobactam, fluoroquinolone)	Other (reduced permeability to antibiotic, based on CARD, it can actually be considered as an efflux pump complex or subunit conferring antibiotic resistance)	RamA (resistance antibiotic multiple) is a positive regulator of AcrAB-TolC.	General Bacterial Porin with reduced permeability to beta-lactams, resistance-nodulation-cell division (RND) antibiotic efflux pump	0.09% (2/2170)
*rsmA*	ARG-CORE	MDR (e.g., diaminopyrimidine, phenicol, fluoroquinolone)	RND efflux	*csrA*; Ribosomal RNA small subunit methyltransferase A	resistance-nodulation-cell division (RND) antibiotic efflux pump	99.91% (2168/2170)
*salB*	ARG-ACC	MDR (e.g., streptogramin, lincosamide, pleuromutilin, streptogramin A)	antibiotic target protection	-	sal-type ABC-F protein	0.05% (1/2170)
*salC*	ARG-ACC	MDR (e.g., streptogramin, lincosamide, pleuromutilin, streptogramin A)	antibiotic target protection	-	sal-type ABC-F protein	1.11% (24/2170)
*srmB*	ARG-ACC	macrolide	antibiotic target protection	ATP-dependent RNA helicase SrmB	Miscellaneous ABC-F subfamily ATP-binding cassette ribosomal protection proteins	0.97% (21/2170)
*vanT* gene in *vanG* cluster	ARG-ACC	glycopeptide	antibiotic target alteration	-	glycopeptide resistance gene cluster, *vanT*	75.62% (1641/2170)
*vanTr* gene in *vanL* cluster	ARG-ACC	glycopeptide	antibiotic target alteration	-	glycopeptide resistance gene cluster, *vanT*	24.33% (528/2170)
*yajC*	ARG-ACC	MDR (e.g., tetracycline, disinfecting agents and antiseptics, glycylcycline, rifamycin, cephalosporin, penam, phenicol, fluoroquinolone, glycopeptide, oxazolidinone)	RND efflux	Sec translocon accessory complex subunit YajC	resistance-nodulation-cell division (RND) antibiotic efflux pump	47.05% (1021/2170)
*ybhF*	ARG-ACC	MDR	ABC efflux	putative multidrug ABC-transporter ATP-binding protein YbhF	-	0.05% (1/2170)
*ybhR*	ARG-ACC	MDR	ABC efflux	putative multidrug ABC transporter permease YbhR	-	7.33% (159/2170)
*ybhS*	ARG-ACC	MDR	ABC efflux	putative multidrug ABC transporter permease YbhS	-	57.47% (1247/2170)
*yheH*	ARG-ACC	MDR	ABC efflux	putative multidrug resistance ABC transporter ATP-binding/permease protein YheH; *bmrA*	-	1.29% (28/2170)
*MATE*_*efflux*_*yeeO*	ARG-CORE	MDR	MATE efflux	Can be shown as *yeeO* by Prokka annotation	-	0.05% (1/2170)
*ACR*_*tran*	ARG-CORE	MDR	ABC efflux	Can be shown as several different names (*mdtcA*, *czcA*, *cusA*, or *bepA*) by Prokka annotation. Should be manually curated to differ from the above genes.	-	99.91% (2168/2170)
**EPs related to AMR** ^a,b^ (Locus tag based on *H. pylori* strain 26695 [NC_000915.1]. Please refer to Table S1 for more information for the reference and gene characteristics.)						
HP0600	ARG-ACC	MDR (e.g., metronidazole, levofloxacin)	ABC efflux	*spaB*	-	62.53% (1357/2170)
HP0605	ARG-CORE	MDR (bilesalt, cefotaxime, ceragenin, clindamycine, clarithromycin, erythromycin, ethidium bromide (EtBr), novobiocin, metal ion, nickel, sodium deoxycholate, tetracycline)	RND efflux	*hefA*; efflux RND transporter outer membane subunit HefA	-	99.68% (2163/2170)
HP0606	ARG-CORE	MDR (bilesalt, cefotaxime, ceragenin, clindamycine, clarithromycin, erythromycin, EtBr, novobiocin, metal ion, nickel, sodium deoxycholate, tetracycline)	RND efflux	*hefB* (alternative name: *acrA* or *mtrC*); efflux RND transporter periplasmic adaptor subunit HefB	-	99.68% (2163/2170)
HP0607	ARG-CORE	MDR (bilesalt, cefotaxime, ceragenin, clindamycine, clarithromycin, erythromycin, EtBr, novobiocin, metal ion, nickel, sodium deoxycholate, tetracycline)	RND efflux	*hefC* (alternative name: *acrB*); efflux RND transporter permease subunit HefC	-	99.63% (2162/2170)
HP0759	ARG-CORE	MDR	MATE efflux	conserved hypothetical integral membrane protein; MATE family efflux transporter	-	99.77% (2165/2170)
HP0791	ARG-CORE	metal (cadmium, zinc)	ABC efflux	*cadA;* heavy-metal translocating P-type ATPase	-	99.59% (2161/2170)
HP0969	ARG-CORE	MDR (e.g., cadmium, metronidazole, nickel, zinc)	RND efflux	*hefF* (alternative name: *czcA1* or *cznA*)	-	99.86% (2167/2170)
HP0970	ARG-CORE	MDR (e.g., cadmium, metronidazole, nickel, zinc)	RND efflux	*hefE* (alternative name: *czcB1* or *cznB*); efflux RND transporter periplasmic adaptor subunit	-	99.77% (2165/2170)
HP0971	ARG-CORE	MDR (e.g., cadmium, metronidazole, nickel, zinc)	RND efflux	*hefD* (alternative name: *cznC*) Note: HefFDE is a homolog of MexA	-	99.77% (2165/2170)
HP1072	ARG-CORE	copper	ABC efflux	*copA*	-	99.72% (2164/2170)
HP1082	ARG-CORE	MDR (erythromycin, etbr, novobiocin, rifampin, and lipopolysaccharide)	ABC efflux	*msbA*	-	99.63% (2162/2170)
HP1091	ARG-CORE	-	MFS efflux	*kgtP*	-	98.57% (2139/2170)
HP1120	ARG-CORE	MDR	ABC efflux	CcmA; Multidrug efflux system ATP-binding protein (NP_208012.1); ABC-type multidrug transport system, ATPase component (COG1131)	-	99.68% (2163/2170)
HP1165	ARG-CORE	tetracycline	MFS efflux	*tetA*	-	99.49% (2159/2170)
HP1174	ARG-CORE	D Glactose (non-drug)	MFS efflux	*gluP*	-	99.68% (2163/2170)
HP1181	ARG-CORE	MDR (e.g., tetracycline, nitroimidazole, fluoroquinolone)	MFS efflux	Multidrug efflux transporter	-	99.95% (2169/2170)
HP1184	ARG-CORE	norfloxacin and ethidium	MATE efflux	NorM; HP1184 family multidrug efflux MATE transporter, conserved hypothetical integral membrane protein; MatE Polysacc_synt_C	-	99.77% (2165/2170)
HP1206	ARG-ACC	MDR (possibly related to metronidazole and levofloxacin resistance)	ABC efflux	*hetA;* multidrug resistance protein (HetA)	-	94.70% (2050/2170)
HP1250	ARG-CORE	-	ABC efflux	Csd5	-	96.96% (2104/2170)
HP1251	ARG-CORE	-	ABC efflux	oligopeptide ABC transporter, permease protein (OppB); microcin C transport system permease protein	-	99.72% (2164/2170)
HP1252	ARG-CORE	-	ABC efflux	OppA	-	99.59% (2161/2170)
HP1327	ARG-CORE	metal (copper, cobalt, zinc cadmium ion)	RND efflux	*hefG* (alternative name: *crdB*); copper resistance outer membrane protein CrdB	-	99.40% (2157/2170)
HP1328	ARG-CORE	metal (copper, cobalt, zinc cadmium ion)	RND efflux	*hefH* (alternative name: *czcB2*); efflux RND transporter periplasmic adaptor subunit	-	99.63% (2162/2170)
HP1329	ARG-CORE	metal (copper, cobalt, zinc cadmium ion)	RND efflux	*hefI* (alternative name: *czcA2, cusA*)	-	99.31% (2155/2170)
HP1487	ARG-CORE	MDR (e.g., novobiocin, deoxycholate, EtBr resistance)	RND efflux (or ABC efflux)	ABC-2 type transport system permease protein	-	99.59% (2161/2170)
HP1488	ARG-CORE	MDR (e.g., novobiocin, deoxycholate, EtBr resistance)	RND efflux (or ABC efflux)	Membrane-fusion protein HlyD family secretion protein	-	99.59% (2161/2170)
HP1489	ARG-CORE	MDR (e.g., novobiocin, deoxycholate, EtBr resistance)	RND efflux (or ABC efflux)	TolC-like outer membrane efflux protein	-	99.59% (2161/2170)
HP1503	ARG-CORE	metal	ABC efflux	cation-transporting ATPase, P-type (*copA*), P-type Cu+ transporter	-	99.77% (2165/2170)
HP1561	ARG-CORE	metal (nickel, copper)	ABC efflux	Iron(III) ABC transporter, periplasmic iron-binding protein (*ceuE*), iron complex transport system substrate-binding protein	-	98.02% (2127/2170)

^a^ The information (including the short description, homolog, and alternative name) and mechanisms of the EP genes were obtained based on previous publications and the KEGG database for *Helicobacter pylori* 26695 [5,35,38,44,47,48,49,50,51,52]. Some of the information on antibiotic targets and alternative names was also presented based on the CARD database [34]. The efflux pumps’ classification was assigned based on previous studies and established databases [47,53]. We have supplemented additional optional information whenever discrepancies were identified between the studies. ^b^ Some of the gene names were described based on the previous studies; therefore, some names might be different than the original annotation of the *H. pylori* 26695 genome in GenBank (NC_000915.1 or NC_018939.1).

**Figure 3:**
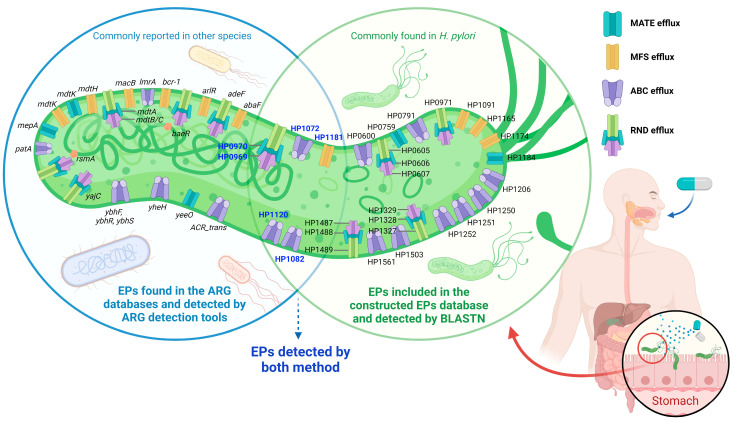
Summary of efflux pumps (EPs) related to AMR detected from the *H. pylori* genome. The green circle contains Eps reported by previous *H. pylori* AMR studies. The sequences of these Eps were collected from *H. pylori* strain 26695 then used as a query for BLASTN against the global *H. pylori* sequence dataset (2170 strains). The result showed that most of these EPs were ARG-CORE. The blue circle contains EPs identified by ARG detection tools and databases. Many of these EPs were found in low numbers of *H. pylori* and were commonly reported in other species of bacteria. This result suggests that some EP genes may have been obtained by HGT via MGE transfer.

Next, the comparison between ARG detection and BLASTN against the constructed 29 EPs database showed that six *H. pylori* EPs could be detected by both ARG detection tools and BLASTN (Figure 3). This result suggests that the ARG databases already contain some EPs that are commonly found in *H. pylori* (ARG-CORE), although the names might be different between databases and *H. pylori* annotations. The combination of these methods showed that *H. pylori* could have at least 50 EPs-related AMR genes. While most of the EPs in the constructed EPs gene database that had detected by BLASTN were already reported in *H. pylori* (Figure 3, green circle), most of the genes detected by ARG detection tools were commonly reported in other species (e.g., *E. coli*). Some genes detected by ARG detection tools were only identified in a few strains, suggesting that these genes might have been obtained by HGT via Mobile Genetic Elements (MGE), possibly from other species living in the gastrointestinal tract (Figure 3, blue circle). 

### 2.6. Global Geographic and Population Distribution of the ‘Set of ARG Commonly Found in the Accessory Genome of H. pylori’ (ARG-ACC)

Understanding the distribution of ARG is essential to illuminate the general epidemiology of ARG patterns in global *H. pylori*. Since all *H. pylori* have ARG because of ARG-CORE, we focused on the distribution of ARG-ACC, which is unique for every strain. We clustered the ARG-ACC genes based on their antibiotic class and identified their distribution based on the geographic location and *H. pylori* population. 

**Figure 4:**
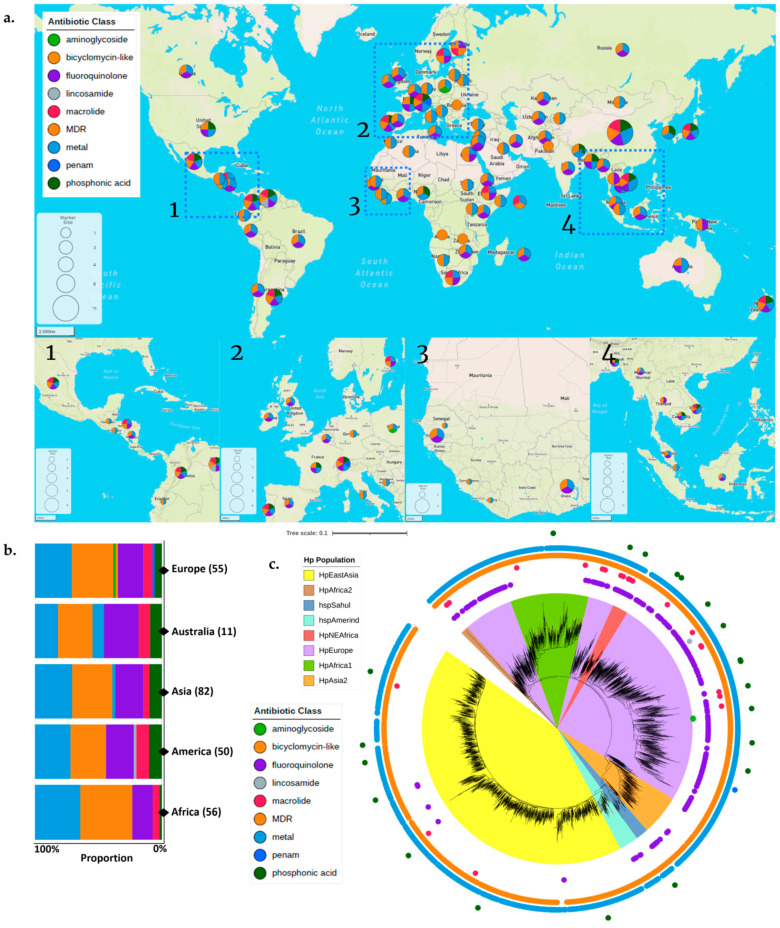
The ARG-ACC distribution based on the geographic location and *H. pylori* population. The ARG-ACC were clustered based on their antibiotic classes. (**a**) The ARG-ACC distribution tended to be similar between countries. Some areas are magnified to show the distribution more clearly. The magnified regions were displayed in correspondence with their respective numerical designations. (**b**) The ARG-ACC proportion for each continent. The proportion tended to be the same for each continent. (**c**) The ARG-ACC distribution is based on the *H. pylori* population. Each dot outside the phylogenetic tree represents the ARG-ACC distribution, with each color representing its antibiotic class.

The geographic assessment showed that the distribution of ARG-ACC genes was almost same between countries. However, we found that some antibiotic classes are unique to a particular country or region (Figure 4a and Figure S6, Spreadsheet S7). These antibiotic classes were the aminoglycosides, bicyclomycin-like, lincosamide, and penam, which were only found in Poland, Finland, Venezuela, and Switzerland, respectively, in our dataset. In addition, genes related to nickel and cobalt resistance were only found in China, Vietnam, and Australia. Overall, while ARG-ACC genes from different antibiotic classes were generally found on all continents, each continent had unique characteristics, e.g., aminoglycoside and lincosamide were only shown in Europe and America, respectively, and the phosphonic acid proportion was lower in Africa compared to the other continents (Figure 4b). 

Next, the assessment of the *H. pylori* population showed that several ARG-ACC in some antibiotic classes seemed evenly distributed, while some were predominantly found in a particular population. The ARG-ACC related to bicyclomycin-like and metal resistance (nickel, cobalt, zinc, and cadmium) could be found in all *H. pylori* populations. In addition, the ARG-ACC related to macrolide and fluoroquinolone resistance was predominantly found in HpEurope, compared to other populations (Figure 4c). 

### 2.7. Association between ARG and Antimicrobial-Resistant Phenotype in H. pylori

Acquiring ARG via HGT may affect the resistance phenotype in bacteria; however, the development of resistance is multifactorial and unique between species of bacteria [54]. We attempted to understand the difference in the presence of ARG between the AMR phenotypes of clinical isolates using antibiogram data from clinical isolates collected from multiple nations. The result suggested no difference in the total ARG between resistant and susceptible strains. However, *H. pylori* resistant to metronidazole and levofloxacin tended to have a higher average total number of ARG compared with the susceptible group (Figure S7). Therefore, this result suggests that higher number of ARG maybe correlate with the *H. pylori* MIC phenotype in certain antibiotics.

Next, we tried to screen and predict other genes as new ARG candidates related to resistance phenotypes by analyzing whole-genome sequencing (WGS). A total of 11, 12, and 11 genes were significantly higher in the resistant group compared with the susceptible group for clarithromycin, metronidazole, and levofloxacin, respectively. Most of these genes were hypothetical proteins (Table 3, Spreadsheet S8). 

These new ARG candidates were analyzed based on the gene presence or absence, which needs careful interpretation. The findings of this method could be confusing if the results showed genes that should theoretically appear in most strains or are commonly found as core or essential genes. Examples of these genes were *topA* and *lolD*. Beginning with the *topA*, our further validation suggested that most strains have a minimum of one *topA* (2168/2170; 99.91%). This result can be partially explained when we understand the input file of the analysis tool. When Prokka finds more than one hit of the same gene within a genome, the General Feature Format (.gff) file output will yield the number for each gene name, which is considered the copy number. In the strains with a single *topA* copy, Prokka’s .gff file will show it as ‘*topA*’, while in the strains with multiple copies, the .gff file will show them numbered, such as ‘*topA*_1′ and ‘*topA*_2′, among others. In this study, we found that the presence of ‘*topA*_*2*′ had a significant difference between resistant and susceptible groups. Therefore, this result suggests that strains with multiple copies of the *topA* gene were significantly more frequent in the resistant group, compared to the susceptible group which consisted mostly of strains with a single *topA* copy. The presence of multiple *topA* in some strains is theoretically possible since this gene has several important roles and can exist in the plasticity region of the type four secretion system (tfs) in *H. pylori* [55,56]. After *topA*, the next example is *lolD*. While the other lipoprotein (Lol) system might not exist in *H. pylori* (e.g., *lolB*), a study just recently reported that *lolD* was present in *H. pylori* as HP0179 [57]. In this study, we found that *lolD* (encoding LolD or LolD-like protein) was present in majority of the strains (2112/2170; 97.33%). In this study dataset, only 9 out of 18 metronidazole-susceptible strains had *lolD*, while 37 out of 43 resistant strains had *lolD*. These results yielded statistically significant differences based on the Scoary algorithm (Spreadsheet S8).

## 3. Discussion

AMR is currently the most significant handicap for the eradication of *H. pylori*. While there has been comprehensive research on the development of AMR related to mutations in well-known genes [5], studies on the global *H. pylori* ARG epidemiology are limited. Our work herein illuminated several challenges in the study of *H. pylori* ARG, including the preferred tools for the detection of ARG in the *H. pylori* genome, the global distribution of *H. pylori* ARG, and the effect of the presence of ARG on the clinical AMR phenotype. 

The first challenge is that there is no standard or recommended tool for ARG detection in *H. pylori*. This challenge needs to be resolved because different tools lead to different outcomes. The discrepancies between the results of recent studies support this finding. For example, a recent study showed that HP1181 was absent from most of their strain dataset [10], while another study showed that this gene was commonly found in all strains in their dataset [12]. While different strain datasets may be another reason for discrepancies, we showed in this study that even within the same dataset, the results could differ when the analysis was performed using different tools. This is because different tools use different databases and methods for ARG detection, with none of them being specific to *H. pylori* [20]. Thus, avoiding the use of just a single tool or database for ARG detection might yield the best results. Currently, combining the results from RGI, AMRFinderPlus, and HMMER using the ResFam profile and the hand selection of ARG from annotation results yields more conclusive results than a single-tool result.

A curation step should then be performed on the combined ARG results. This curation step aims to determine whether the genes are present and minimize false-positive results. Herein, we proposed an easy method, comparing the ARG detection results with the annotation results. This method also provided an advantage in that it led to the discovery of undetected ARG. While the curation step is viable and favored, especially to validate the results from the BLAST-matches-based method, it may be challenging for homology modeling, especially the HMM screening method. This is because the curation method is based on the nucleotide sequence, while homology modeling is often based on the amino acid perspective, which can sometimes be relatively different. Nonetheless, the curation method is still required for more accurate results. Many tools or databases were also curating their results before publishing the final data, for example, the hand curation of the HMM profile database that was performed by the developer of ResFam [26]. In addition, it may be difficult to remove the possibility of duplication genes resulting from sequencing, assembly, and/or annotation artifacts. Therefore, future studies should be careful to identify the gene copy number of ARG, especially when the sequence quality is low or unknown. 

An interesting finding in this study after hand curation was that several ARG could be found in most strains. We categorized these ARG as the core genome, called ARG-CORE. An example of this group is genes related to EPs, such as *msbA*. This finding is supported by previous studies stating that several ARG were also found in most of their strain datasets [10,12]. 

In addition, our findings regarding some EPs could be found in almost all the strains, which was in line with a recent study conducted in Shanghai [10]. While previous studies were mostly conducted in a limited region with a limited sample, this study yielded broader knowledge regarding ARG and EPs-related AMR at the global level.

The second challenge is the lack of global epidemiological data on ARG distribution. We reconciled this challenge by presenting the current ARG data based on the geographic location and the *H. pylori* population. We focused on the ARG-ACC rather than the ARG-CORE. For the geographic distribution, the ARG-ACC distribution seemed to be nearly similarbetween geographic locations. The differences between the geographic locations arose because certain genes can be found in a few strains within a certain region. As for the distribution based on the *H. pylori* population, we found that certain ARG from some antibiotic classes could be mainly found in certain populations compared to others. These findings suggest that the dissemination of certain ARG might be related not only with geographical location, but also with *H. pylori* population. This is possible because different populations may have different genetic characteristics, leading to different compatibilities and capabilities for HGT, for example, by the Mobile Genetic Elements [58].

Finally, the last challenge is the effect of the presence of ARG on the clinical AMR phenotype. Our analysis suggested that ARG may not significantly affect the AMR status of *H. pylori*. However, we found that a higher total number of ARG within the genome may lead to a higher MIC phenotype. Previous experimental studies for particular *H. pylori* ARG support this finding. For example, a study showed that *hp1181* correlated with the active efflux phenotype of multidrug-resistant *H. pylori* isolates [27]. 

In addition, we tried to look for ARG candidates simply by comparing the presence and absence of the genes between resistant and susceptible strains. Some genes that were found significantly more frequently in the resistant strains compared to the susceptible strains might provide a starting point for future studies of *H. pylori* antimicrobial resistance. One example of such gene is *cptA*. While previous studies showed that *cptA* may or may not be correlated with the AMR phenotype of a particular antibiotic [59,60], a study of the effect of *cptA* on clarithromycin resistance in *H. pylori* remains necessary. This gene was found as part of the toxin–antitoxin system (TAS) in *H. pylori* [61], and it is well-known that the TAS has a role in maintaining and disseminating antimicrobial resistance in Gram-negative bacteria [62]. In addition, we reported several rare genes usually present in other species for the first time. For example, *mecA* is well-known to be correlated with penicillin-like antibiotic resistance (e.g., methicillin or penicillin) in some species, especially methicillin-resistant *Staphylococcus aureus* (MRSA) [63]. The presence of a highly similar sequence in *H. pylori* suggests that this gene may be inserted via HGT during the transfer of MGE. Many MGE are well-known to have a role in the dissemination of ARG via HGT, either intra- or inter-species [54,64,65]. Since there is no global-level study of MGE in *H. pylori*, their role in *H. pylori* remains unclear, thus, leading to new opportunities for future study. 

There are a number of limitations associated with this study that should be taken into account for future research in *H. pylori* AMR, ARG, and EPs-related resistance studies. First, different annotation tools might yield different results, and we did not compare the results based on other tools such as RAST or PGAP. Prokka annotation itself should be performed by using several good complete genomes as the reference to obtain better results. Second, the distribution number was obtained based on the presence or absence of genes by ABRICATE, while different tools might yield slightly different total number results. We tried to compare the results by using: (1) ABRICATE, (2) BLASTN of nucleotide queries against genome assemblies, (3) BLASTP (protein) of amino acid queries against gene protein sequences, and (4) grep by using keywords (the name of the genes for the .fna, .faa, and .gff files of the Prokka output). The results showed almost similar results for the total number of positive strains (i.e., strains that harbour ARG or EPs-related AMR); however, different results for the gene copy number. Thus, any of these methods are acceptable for checking the presence or absence of the genes; however, BLASTN or BLASTP might be better to detect more accurate gene copy numbers with high similarity sequences. In this study, we focused on analyzing the gene presence or absence per strain, and we used ABRICATE for identifying the epidemiology distribution of each gene since ABRICATE yields the most convenient output result and it can use BLASTN or BLASTP. Third, although Prokka may detect multiple copies of a gene, there seemed to be some differences between those copies since our homology search could usually only find 1 or 2 copies. For example, Prokka detected five copies of *topA* in strain HP ISR15; however, the homology result for this strain showed only two hits (in all searches and filters). Future studies should assess this difference to yield more conclusive results for *topA*-related AMR studies in *H. pylori*. Finally, the new ARG candidate was identified simply using the presence/absence of genes with a relatively small number of strains, even though the data were collected from several countries. Thus, it might not be enough to represent the general *H. pylori* condition. Nevertheless, the findings in this study need to be confirmed by mutation analysis of well-known antibiotic target alteration genes, as this is the primary mechanism of AMR phenotype development, as reported by previous studies [5,66,67]. A higher number of strain datasets with antibiogram data, as well as further in-depth analysis, for example, by genome-wide association studies at a global level (with the *H. pylori* population constructed by fineSTRUCTURE), are necessary to provide more valuable information regarding the impact of ARG on the phenotypes of clinical isolates. 

This study leads to many opportunities for future *H. pylori* AMR investigations. We have provided a list of the ARG that can be used to construct a new database for *H. pylori* ARG and EPs-related AMR detection from the genome sequence. Developing a tool that detect ARG and mutations with the database curated for *H. pylori* is necessary. Furthermore, most of the genes reported as ARG in this study have a lack of experimental validation in *H. pylori*. Further experimental studies on these genes are appealing to be conducted by future study.

## 4. Materials and Methods

### 4.1. WGS Dataset Collection

A total of 2185 WGS data points were retrieved. Except for isolates from Indonesia, Vietnam, and Bangladesh, all data in this investigation were obtained directly from public databases and downloaded consecutively up to 22 June 2021 (DDBJ/EnteroBase/EMBL/GenBank/Patric) [68,69,70,71,72,73]. Genomes were reported from more than 60 countries on 6 continents worldwide. After removing duplications, we performed quality-control filtering of results via QUAST version 5.0.2 [74]. Following the RefSeq criteria, all genomes with fragmented assemblies (L50 > 500, N50 < 5000, or consisting of more than 2000 contigs), too large/too small genome lengths (shorter than 807.5 kbp or longer than 2422.5 kbp), and format abnormalities (sequence containing non-ACGTN characters) were excluded [75]. Finally, 2170 WGS were selected for further genomic analysis (Spreadsheet S9).

### 4.2. WGS Annotation and H. pylori Population Construction

All WGS data were annotated with Prokka version 1.14.6 to identify WGS coding sequences [76]. We used seven *H. pylori* housekeeping genes to identify the populations according to PubMLST [77]. We extracted the genes using NCBI Blast+ version 2.12.0 [78], then aligned them using MAFFT version 770 [78]. We used the best method based on the model finder in IQ-TREE version 2.0.6 [79].

### 4.3. ARG and EP Detection

Several in silico analysis tools and well-known databases were applied to increase the sensitivity of ARG detection. The nucleotide fasta files (.ffn) from the Prokka output were used to run each process. The tools and databases used were as follows:

- ABRICATE v1.0.1 (BLAST-matches-based method that applies BLASTN) [14], against several ARG databases:
  (a) ARG-ANNOT (v5, last update 2019) [15]
  (b) CARD (Narch 2020 update) [16,17]
  (c) MEGARes (v2.0, last update 2020) [18]
  (d) ResFinder (last update 2019) [19]
  Parameter settings: minimum coverage and identity, 90.
- ResFinder v4.2.3 (BLAST-matches-based method) [19], against the ResFinder database (last update October 2022).
  Parameter settings: minimum coverage and identity, 90.
- The Resistance Gene Identifier (RGI) v6.0.1 (homology and SNP models) [17], against the CARD database (v3.2.5, last update 2022) [34].
  Parameter settings: default, including ‘strict’ and ‘perfect’ only.
- AMRFinderPlus v3.11.2 (combination of BLAST-matches, HMM screening, and other improvements) [24], against a set of databases that combine:
  (a) Pathogen Detection Reference Gene Catalog (https://www.ncbi.nlm.nih.gov/pathogens/refgene/#, accessed on 22 July 2021)
  (b) Pathogen Detection Reference HMM Catalog (https://www.ncbi.nlm.nih.gov/pathogens/hmm/, accessed on 22 July 2021)
  (c) Bacterial Antimicrobial Resistance Reference Gene Database (https://www.ncbi.nlm.nih.gov/bioproject/PRJNA313047, accessed on 22 July 2021)
  (d) NCBIfam-AMRFinder (https://ftp.ncbi.nlm.nih.gov/hmm/NCBIfam-AMRFinder/latest/, accessed on 22 July 2021)
  The aforementioned information regarding databases is derived automatically from ‘AMRFinderFinderPlus’ as the default source. Our access to these database was not manually one by one; rather, we obtained it by querying the ‘AMRFinderFinderPlus’ database as a unified resource (for more information please visit: https://github.com/ncbi/amr/wiki, accessed on 21 December 2022). Parameter settings: default parameter, add ‘—plus’ flag, and three file types as input (.fna, .faa, and .gff).
- HMMER v3.3.2 (http://hmmer.org/ (accessed on 21 December 2022) Howard Hughes Medical Institute) (The Hidden Markov model (HMM)-based method), against the ResFam Profile Database [25,26] (v1.2.2, last update 2018).
  Parameter settings: --incE, 1 × 10^−10^; -E, 1 × 10^−10^; bit score ≥100, ≥200, ≥250, ≥300, ≥400, and ≥500. We used the Prokka (.faa) output file to run HMMER. We excluded the results if they contained ‘antibiotic target alteration’ or unclear information. After hand curation against the Prokka annotation results, we excluded genes that should not belong to ARG.

Please refer to the Supplementary Spreadsheets for the results of each detection method. In addition to the BLAST-matches-based method, the CLC Genomics Workbench 22 (QIAGEN, Aarhus, Denmark) was used to align the MFS sequences from the CARD or MEGARes databases, as well as MFS sequences from some of the representative strains.

As for Eps’ detection, we constructed a reference-based sequence database of 29 EPs related to AMR or MDR. The sequences of the EP genes were retrieved from *H. pylori* strain 26695 (NC_000915.1). The presence/absence detection was performed by using ABRICATE v1.0.1 with the minimum identity and coverage of 80. The results were considered as a duplication if they were found within the same contig with an overlapping region. The duplicate results were removed, retaining only the genes with the highest sequence identity and coverage. 

### 4.4. Curation of ARG Detection Results

The curation was carried out by matching the results of the identified ARG(s) with the results of the Prokka annotation. After combining the genes identified by all the ARG detection tools (Methods 4.3), we identified whether these genes could initially be detected in Prokka by screening whether the detected ARG names were also detected in the Prokka annotation. If the genes linked to the keyword were undetected, we confirmed their absence by performing BLAST from the undetected ARG reference sequence against all the sample fasta files, with a minimum identity and coverage of 90. Finally, we excluded all the false-positive detection results and summarized them as the final results after curation.

### 4.5. Finding Additional ARG from Prokka

We identified the additional ARG not detected by the ARG detection tools by extracting genes containing any of the following keywords in their product: ‘resistant/resistancy/resistance/drug/antibiotic/antimicrobial’.

### 4.6. Protein Model Analysis

We used several tools to obtain the results. Phyre2 was used to infer the similarity between *H. pylori* MFS and previously reported proteins with similar structures [28]. AlphaFold2 was used to build the MFS antibiotic EP model in *H. pylori* [33]. We chose the best model from five optional predicted models of AlphaFold2 based on its recommendation (Figure S4). The selected model was then used as the input for the protein structure comparison against the ‘drug efflux protein—MFS Transporter’ reported from *E. coli* (PDB DOI: 10.2210/pdb3WDO/pdb). This PDB was chosen because it showed the highest coverage, identity, and confidence in the Phyre2 results. The protein structure comparison was performed by SWISS-MODEL (showing the structure as well as the ensemble consistency and variance) and MASS (calculating the average RMSD) [31,32].

### 4.7. Analysis of the ARG Presence Status with the AMR Phenotype and Detection of New ARG Candidates

A total of 164 antibiogram data points (MIC value and resistance status following the EUCAST criteria) from 61 strains were retrieved from public databases [80,81,82] and our group experimental data collection [83,84,85]. These antibiogram data had WGS and were already included in 2185 WGS datasets. Antibiogram data were available for metronidazole, clarithromycin, and levofloxacin (Spreadsheet S10). We used the total number of ARG, including their copy numbers. The average total gene number was used to compare the phenotype status (resistant/susceptible) of the *H. pylori* clinical isolates.

The identification of new ARG candidates was carried out using Scoary v1.6.16. Initially, we constructed the pan-genome from the strains with antibiogram data using Roary v3.12.0 (parameter settings: -e --mafft -i 50 -cd 50). The gene presence/absence file was then used together with the susceptible/resistant information for each antibiotic as the input data for Scoary (with default parameters). We only considered genes with a naive *p*-value < 0.05, and the genes should present a minimum of 75% (ratio 1:3) more often in the resistant group than in the susceptible group.

## 5. Conclusions

In this study, we addressed several challenges in the global epidemiology of ARG and EPs-related AMR in *H. pylori*. Beginning with tools and databases for ARG detection, our analysis showed that combining several tools and their databases (for instance, RGI, AMRFinderPlus, and the ResFam profile), followed by the manual selection of ARG from the annotation results, yielded more conclusive results than the use of a single tool or database. Combining the final results, the geographic assessment showed that the distributions of the ARG and EPs-related AMR genes were almost the similar between countries, with some genes from certain antibiotic classes being unique to a particular country or continent. The same tendency could be observed in the distribution based on *H. pylori* populations. Some ARG antibiotic classes tended to be found in a particular population. Finally, we showed that the presence of ARG may not directly affect with the sensitive/resistant phenotype but may correlate with the MIC phenotype, and this remains to be further investigated. In the future, the development of a tool that focuses on detecting ARG in *H. pylori* is needed. This tool should contain an updated ARG database based on the *H. pylori* studies and should be able to detect not only the presence or absence of ARG but also mutations related to the AMR phenotype in well-known essential genes. 

## Figures and Tables

**Table 3:** Genes that were significantly present in the resistant strains compared to the susceptible strains.

Gene Annotation (Gene Name or Non-Unique Gene Name)	Total Present in Resistant Strains, pr (pr/r; %)	Total Present in Susceptible Strains, ps (ps/s; %)	Naive *p*-Value
**Clarithromycin** **(*n* = 61, r = 35, s = 26)**			
hypothetical protein	10 (10/35; 28.57)	0 (0/26; 0.00)	0.003
phosphoethanolamine transferase CptA (*cptA*_1)	18 (18/35; 51.43)	5 (5/26; 19.23)	0.016
hypothetical protein	8 (8/35; 22.86)	0 (0/26; 0.00)	0.016
hypothetical protein	7 (7/35; 20.00)	0 (0/26; 0.00)	0.017
hypothetical protein	19 (19/35; 54.29)	6 (6/26; 23.08)	0.019
phosphoethanolamine transferase CptA (*cptA*_2)	17 (17/35; 48.57)	5 (5/26; 19.23)	0.030
hypothetical protein	11 (11/35; 31.43)	2 (2/26; 7.69)	0.030
DNA topoisomerase 1 (*topA*_2)	11 (11/35; 31.43)	2 (2/26; 7.69)	0.030
hypothetical protein	9 (9/35; 25.71)	1 (1/26; 3.85)	0.034
hypothetical protein	13 (13/35; 37.14)	3 (3/26; 11.54)	0.038
hypothetical protein (*recF*_1)	14 (14/35; 40.00)	4 (4/26; 15.38)	0.049
**Metronidazole** **(*n* = 61, r = 43, s = 18)**			
lipoprotein-releasing system ATP-binding protein LolD	37 (37/43; 86.05)	9 (9/18; 50.00)	0.007
hypothetical protein	17 (17/43; 39.53)	1 (1/18; 5.56)	0.012
hypothetical protein	16 (16/43; 37.21)	1 (1/18; 5.56)	0.013
trifunctional nucleotide phosphoesterase protein YfkN (*yfkN*)	41 (41/43; 95.35)	13 (13/18; 72.22)	0.020
hypothetical protein (*SIRT5*_2)	10 (10/43; 23.26)	0 (0/18; 0.00)	0.026
hypothetical protein	10 (10/43; 23.26)	0 (0/18; 0.00)	0.026
apolipoprotein N-acyltransferase (*Int*)	34 (34/43; 79.07)	9 (9/18; 50.00)	0.033
hypothetical protein	20 (20/43; 46.51)	3 (3/18; 16.67)	0.042
hypothetical protein (*gspA*)	40 (40/43; 93.02)	13 (13/18; 72.22)	0.042
hypothetical protein	9 (9/43; 20.93)	0 (0/18; 00)	0.047
hypothetical protein	9 (9/43; 20.93)	0 (0/18; 00)	0.047
hypothetical protein (*hsdM*)	9 (9/43; 20.93)	0 (0/18; 00)	0.047
**Levofloxacin** **(*n* = 42, r = 12, s = 30)**			
chromosome partition protein Smc (*smc*)	4 (4/12; 33.33)	1 (1/30; 3.33)	0.018
hypothetical protein	4 (4/12; 33.33)	1 (1/30; 3.33)	0.018
hypothetical protein	4 (4/12; 33.33)	1 (1/30; 3.33)	0.018
hypothetical protein	4 (4/12; 33.33)	1 (1/30; 3.33)	0.018
hypothetical protein	4 (4/12; 33.33)	1 (1/30; 3.33)	0.018
hypothetical protein	3 (3/12; 25.00)	0 (0/30; 0.00)	0.019
transcription-repair-coupling factor (*mfd*)	3 (3/12; 25.00)	0 (0/30; 0.00)	0.019
peptide deformylase 1 (*def*)	3 (3/12; 25.00)	0 (0/30; 0.00)	0.019
competence protein ComM (*comM*)	3 (3/12; 25.00)	0 (0/30; 0.00)	0.019
hypothetical protein	3 (3/12; 25.00)	0 (0/30; 0.00)	0.019
flagellar basal-body rod protein FlgG (*flgG*_2)	3 (3/12; 25.00)	0 (0/30; 0.00)	0.019

*n*, Total number of strains; r, total number of resistant strains; s, total number of susceptible strains; pr, total present in resistant strains; ps, total present in susceptible strains.

## Data Availability

All the extracted EPs genes are available at: http://sayer.nig.ac.jp/kirill/Helicobacter-pylori-Global-Antimicrobial-Resistance-Gene-Study--Supplementary-Data/Helicobacter-pylori-Efflux-Pump-gene-sequences.zip (accessed on 1 May 2023).

## Supplementary Materials

The following supporting information can be downloaded at: https://www.mdpi.com/article/10.3390/antibiotics12071118/s1, Figure S1: Results’ comparison of different parameter settings for minimum identity and coverage. Figure S2: Comparison of nucleotide sequences of the Major Facilitator Superfamily (MFS) efflux pump family retrieved from the CARD database, the MEGARes database, and representative strains (UM067, BGD53, and MS203). Figure S3: An example of an RGI finding that is annotated as a hypothetical protein by Prokka. Figure S4: Protein modeling for the *H. pylori* MFS efflux pump family and its comparison against the ‘drug efflux protein—MFS Transporter’ reported in *E. coli*. Figure S5: The ARG-ACC distribution based on the geographic location. Figure S6: Comparison and trends of ARG numbers between resistant and susceptible *H. pylori* clinical isolates and their MIC data. Table S1: EPs related to AMR reported in *H. pylori*. Most of these genes were already mentioned by recent original and review studies [10,47]. The EPs gene name, length, and GC content were retrieved from *H. pylori* strain 26695 (NC_000915.1). Spreadsheet S1: Global ARG identification results from ABRICATE using several ARG databases. Spreadsheet S2: Global ARG identification results from ResFinder. Spreadsheet S3: Global ARG identification results from RGI–CARD. Spreadsheet S4: Global ARG identification results from AMRFinderPlus. Spreadsheet S5: Hand curation of HMMER against the HMM ResFam Profile. Spreadsheet S6: Hand curation of all results against Prokka results. Spreadsheet S7: Global distribution of ARG-ACC. Spreadsheet S8: ARG candidates by Scoary analysis. Spreadsheet S9: Genome metadata. Spreadsheet S10: Clinical implication metadata.
