# Supplementary material for: Global Antimicrobial Resistance Gene Study of Helicobacter pylori: Comparison of Detection Tools, ARG and Efflux Pump Gene Analysis, Worldwide Epidemiological Distribution, and Information Related to the Antimicrobial-Resistant Phenotype
Source: Antibiotics (Basel). 2023 Jun 28;12(7):1118. doi: 10.3390/antibiotics12071118 (PMC10376887; doi:10.3390/antibiotics12071118)
Supplement: Supplementary file 1 [file antibiotics-12-01118-s001.zip › Supp Information - ARG Study - Antibiotics.pdf]

antibiotics

MDPI

Supplementary Materials

Global Antimicrobial Resistance Gene Study of *Helicobacter pylori*: Comparison of Detection Tools, ARG and Efflux Pump Gene Analysis, Worldwide Epidemiological Distribution, and Patient Clinical Treatment Implications

Ricky Indra Alfaray[1,2], Batsaikhan Saruuljavkhlan[1], Kartika Afrida Fauzia[1,2,3], Roberto C. Torres[4], Kaisa Thorell[5], Selva Rosyta Dewi[1,2], Kirill A. Kryukov[6], Takashi Matsumoto[1], Junko Akada[1], Ratha-korn Vilaichone[7,8,9,10], Muhammad Miftahussurur[2,10,*], Yoshio Yamaoka[1,10,11,12*]

[1] Department of Environmental and Preventive Medicine, Faculty of Medicine, Oita University, 1-1 Idaigaoka, Hasama-machi, Yufu, Oita 879-5593 Japan; rickyindraalfaray@gmail.com (RIA); saruuljavkhlan@yahoo.com (SB); kartikafauzia@gmail.com (KAF); selvard17@gmail.com (SRD); tmatsumoto9@oita-u.ac.jp (TM); akadajk@oita-u.ac.jp (JA); yyamaoka@oita-u.ac.jp (YY.)
[2] Helicobacter pylori and Microbiota Study Group, Institute of Tropical Disease, Universitas Airlangga, Surabaya, East Java, 60286, Indonesia; muhammad-m@fk.unair.ac.id
[3] Department of Public Health and Preventive Medicine, Faculty of Medicine, Universitas Airlangga, Surabaya, 60132, Indonesia
[4] The Center for Microbes, Development and Health, Key Laboratory of Molecular Virology and Immunology, Institut Pasteur of Shanghai, Chinese Academy of Sciences, Shanghai, 200031, PR China; rtorres@ips.ac.cn (RCT);
[5] Department of Chemistry and Molecular Biology, Faculty of Science, University of Gothenburg, Gothenburg, 405 30, Sweden; kaisa.thorell@gu.se (KT.)
[6] Biological Networks Laboratory, Department of Informatics, National Institute of Genetics, Mishima, Japan
[7] Gastroenterology Unit, Department of Medicine, Faculty of Medicine, Thammasat University Hospital, Khlong Nueng 12120, Pathumthani, Thailand; vilaichone@hotmail.co.th (RV.)
[8] Digestive Diseases Research Center (DRC), Thammasat University, Khlong Nueng 12121, Pathumthani, Thailand
[9] Department of Medicine, Chulabhorn International College of Medicine (CICM), Thammasat University, Khlong Nueng 12121, Pathumthani, Thailand
[10] Division of Gastroentero-Hepatology, Department of Internal Medicine, Faculty of Medicine, Dr. Soetomo Teaching Hospital, Universitas Airlangga, Surabaya 60286, Indonesia
[11] The Research Center for GLOBAL and LOCAL Infectious Diseases (RCGLID), Oita University, Oita, 870-1192, Japan
[12] Department of Medicine, Gastroenterology and Hepatology Section, Baylor College of Medicine, Houston, Texas, 77030, USA.

* Correspondence: Y.Y. yyamaoka@oita-u.ac.jp ; Tel.: +81-97-586-5740; Fax: +81-97-586-5749, +81-97-586-5711; M.M. muhammad-m@fk.unair.ac.id; Tel.: +62-31-502-3865; Fax: +62-31-502-3865

Supplementary Figures

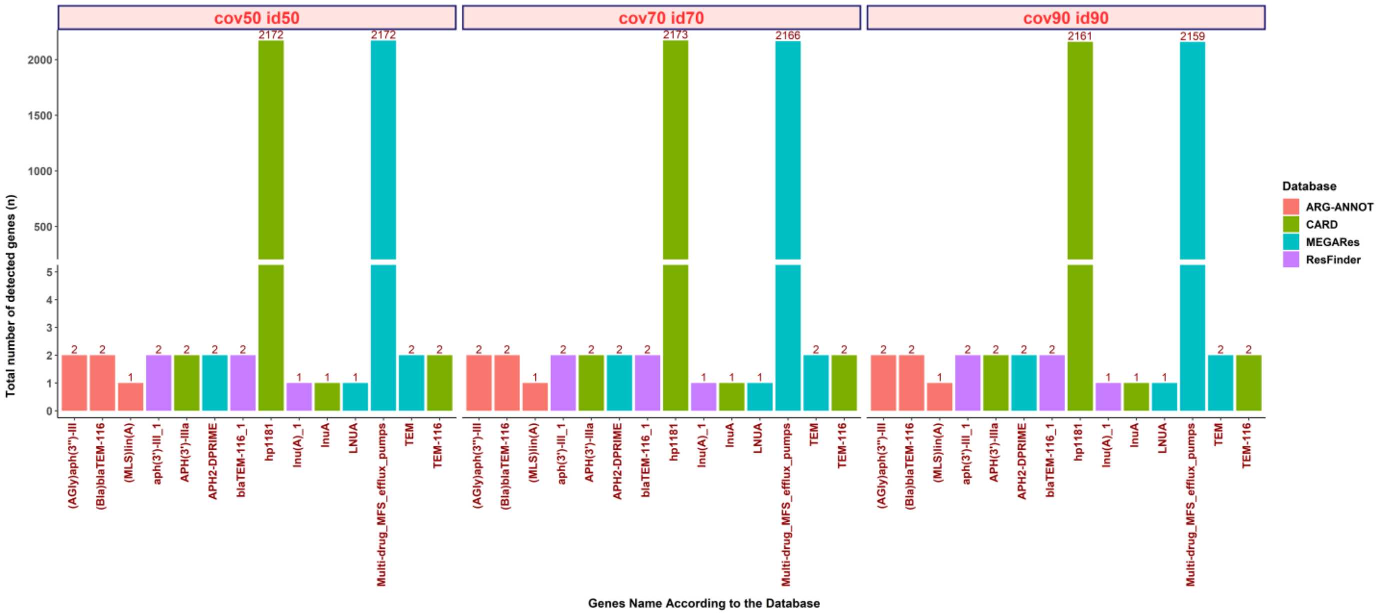

Figure S1. Results comparison of different parameter settings for minimum identity and coverage. The figure shows the number of genes detected instead of the number of strains harboring the gene.

a.

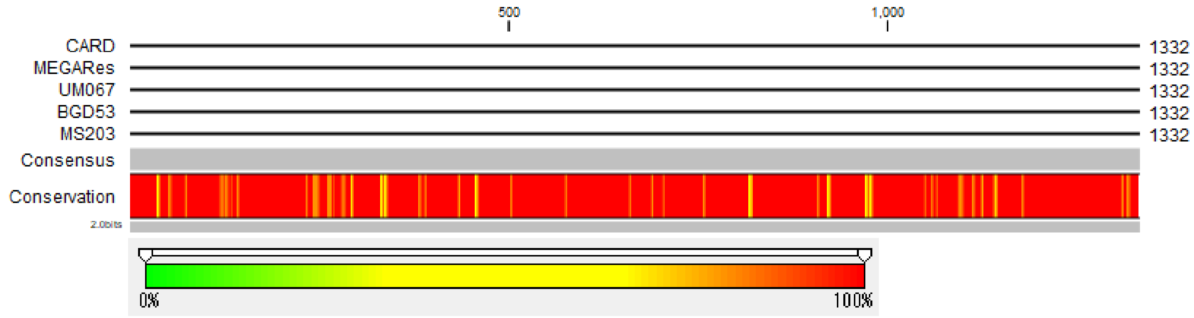

b.

		1	2	3	4	5
CARD	1		100.00	96.25	95.35	94.74
MEGARes	2	100.00		96.25	95.35	94.74
UM067	3	96.25	96.25		95.50	94.52
BGD53	4	95.35	95.35	95.50		95.12
MS203	5	94.74	94.74	94.52	95.12	

Figure S2. Comparison of nucleotide sequences of the Major Facilitator Superfamily (MFS) efflux pump family retrieved from the CARD database, the MEGARes database, and representative strains (UM067, BGD53, and MS203). a. Alignment showing that all the sequences share high similarity and conservation. **b.** Analysis showing that all sequences share >90% identity with each other, suggesting that they belong to the same gene.

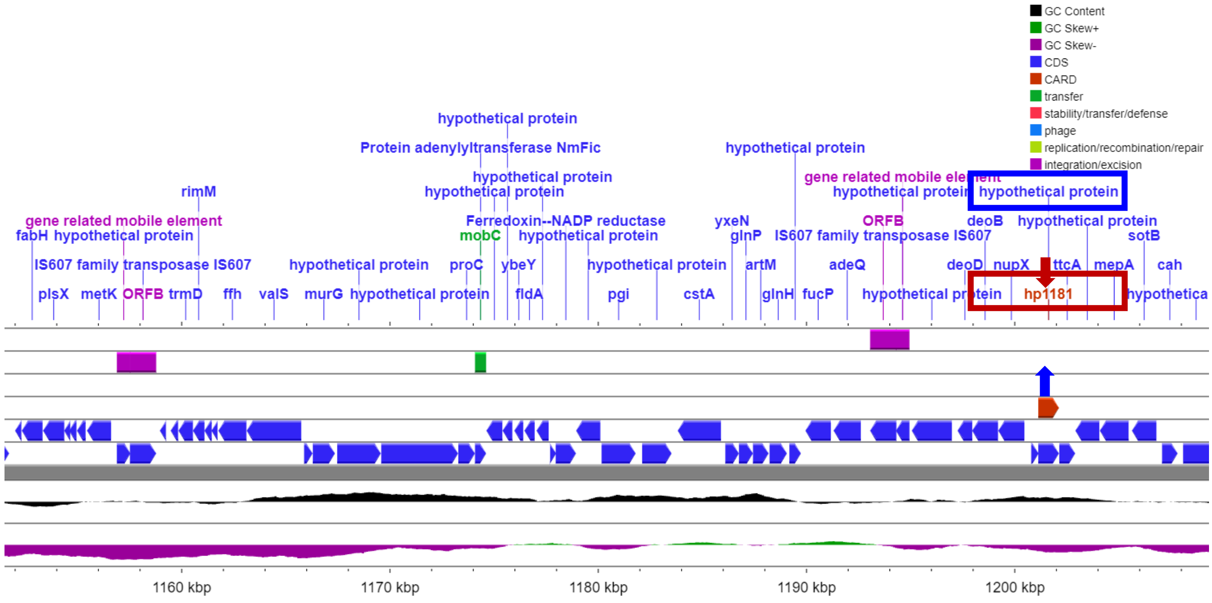

Figure S3. An example of an RGI finding that is annotated as a hypothetical protein by Prokka. The blue box and arrow represent the hypothetical protein annotated by Prokka, while the red arrow and box represent *hp1181*, which RGI identified.

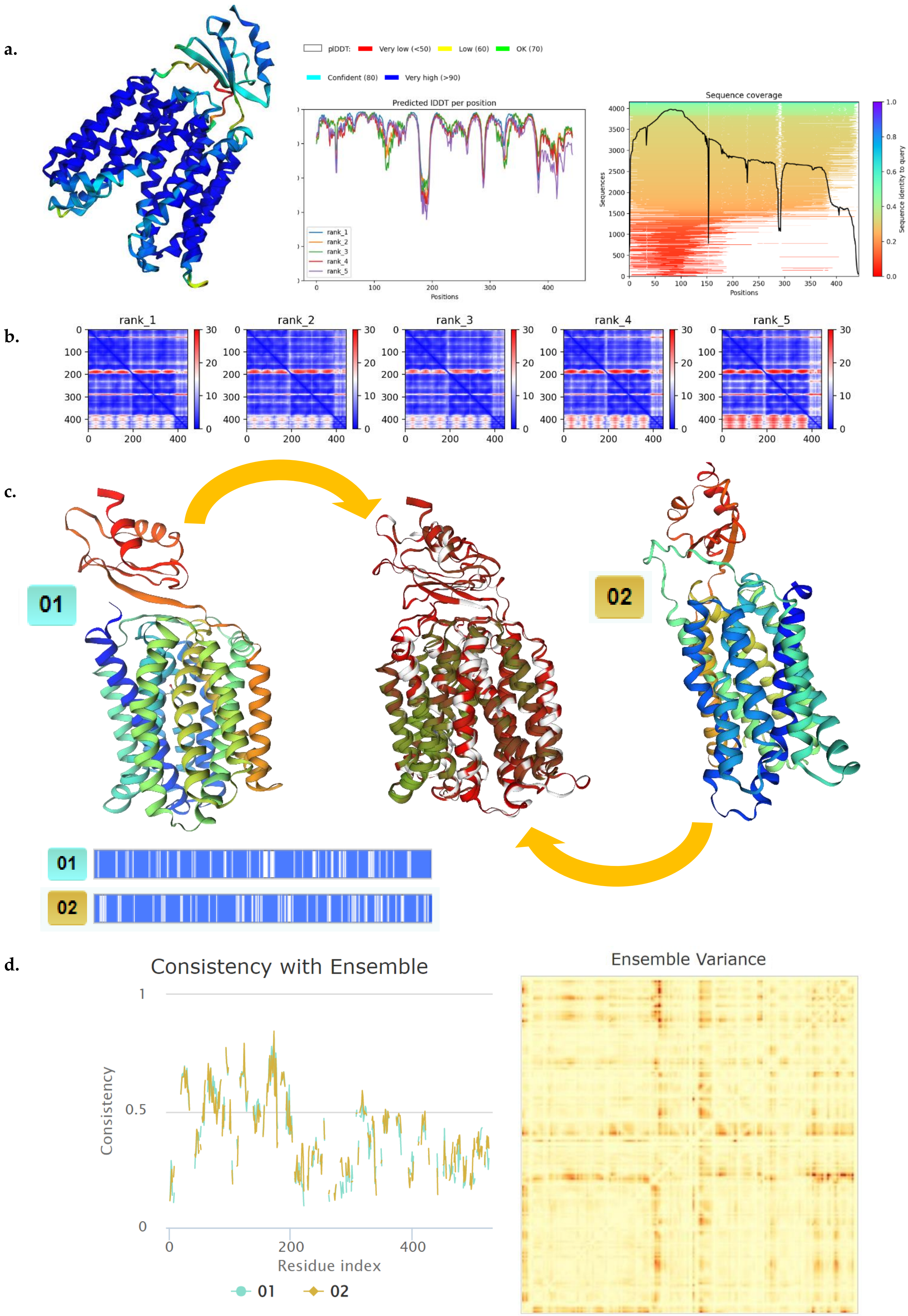

a.
pIDDT: Very low (<50) Low (60) OK (70)
Confident (80) Very high (>90)
Predicted IDDT per position
rank_1
rank_2
rank_3
rank_4
rank_5
Positions
Sequence coverage
Sequences
Positions
Sequence identity to query
b.
rank_1
rank_2
rank_3
rank_4
rank_5
c.
01
02
d.
Consistency with Ensemble
Consistency
Residue index
01
02
Ensemble Variance

Figure S4. Protein modeling for the *H. pylori* MFS efflux pump family and its comparison against 'drug efflux protein - MFS Transporter' reported in *E. coli*. a. The protein model of MFS (hp1181) is commonly found in the global *H. pylori* dataset and the protein modeling data by AlphaFold2. *Left chart:* The sequence identity between the constructed model and the query. *Right chart:* Prediction of the Local Distance Difference Test (IDDT) between five models constructed by AlphaFold2 showing that the overall model is similar. **b.** Predicted Aligned Error (PAE) between five models constructed by AlphaFold2 indicating that AlphaFold2 predicts well-defined relative positions and orientations for all the predicted models. We used the best model based on the AlphaFold2 recommendation as the input against *E. coli* MFS for running SWISS-MODEL and MASS. **c.** Protein model comparison result. *Left*: (01) Protein model of *E. coli* MFS (PDB DOI: 10.2210/pdb3WDO/pdb). *Right:* (02) Protein model of *H. pylori* MFS. *Middle*: Superimposition of the two proteins. The color represents the ensemble consistency between the two proteins after alignment (white, gaps; green to red, higher to lower consistency). *Bottom*: Alignment map showing that most of the protein sequences could be merged because they have equal length, structure, or high sequence identity (blue, sequence that can be aligned and shown in the superimposed structure). **d.** Consistency with Ensemble. *Left*: Consistency with Ensemble shown per residue index; some regions have high consistency. *Right*: The Ensemble variance showing a few off-diagonal 'blocks,' which mostly have low domain movement events.

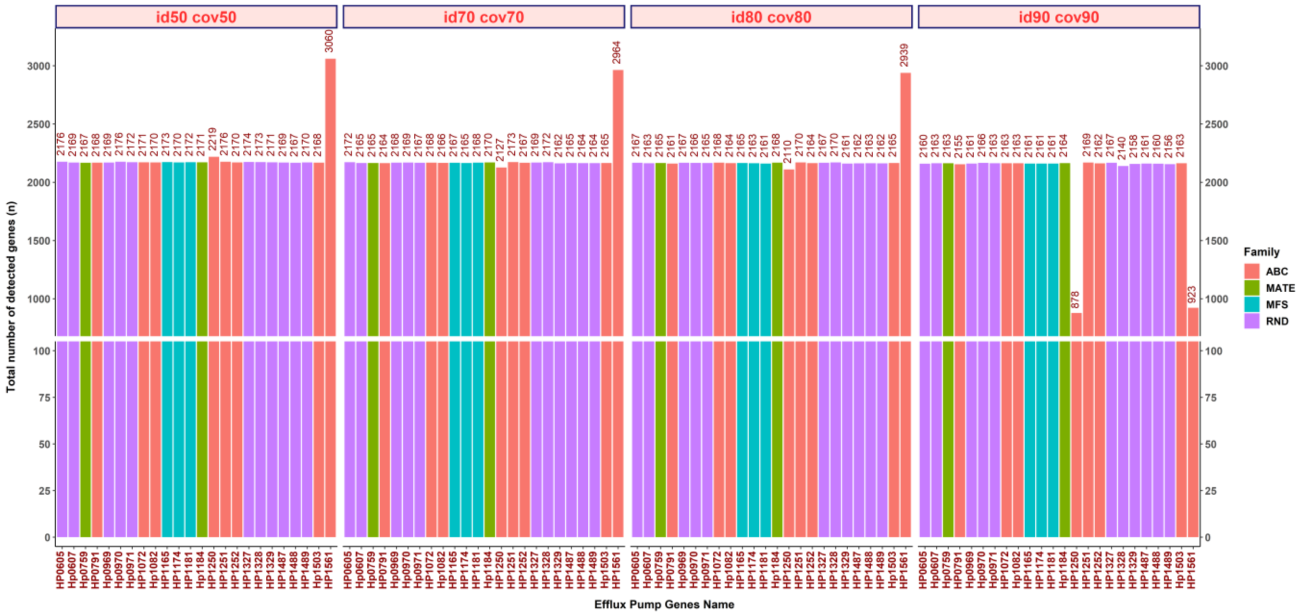

Figure S5. Results comparison of different parameter settings for minimum identity and coverage to detect EPs. The figure shows the number of genes detected instead of the number of strains harboring the gene.

a.

b.

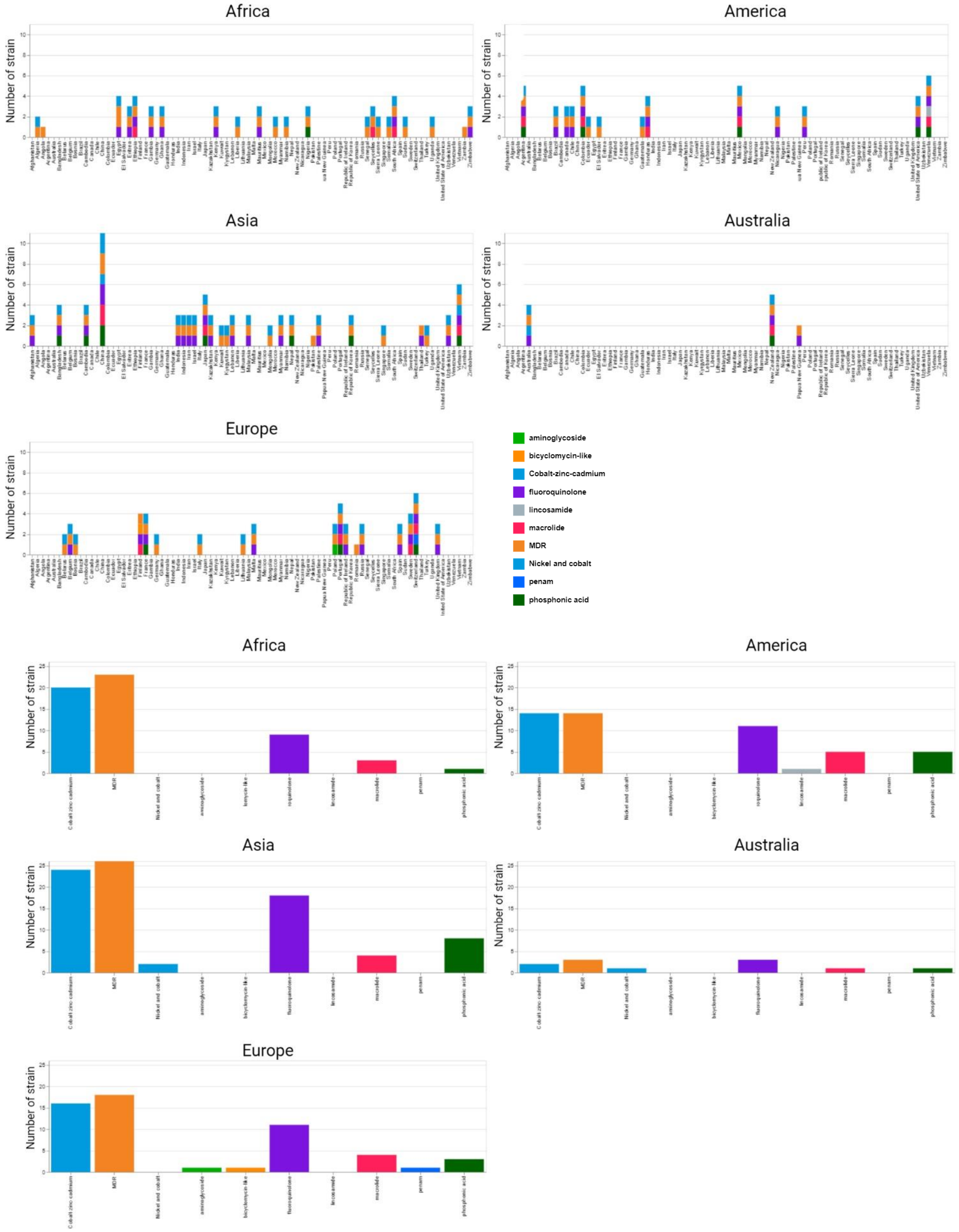

Figure S6. The SA-ACPY distribution based on the geographic location. a. Distribution based on the country. **b.** Distribution on each continent based on the antibiotic class.

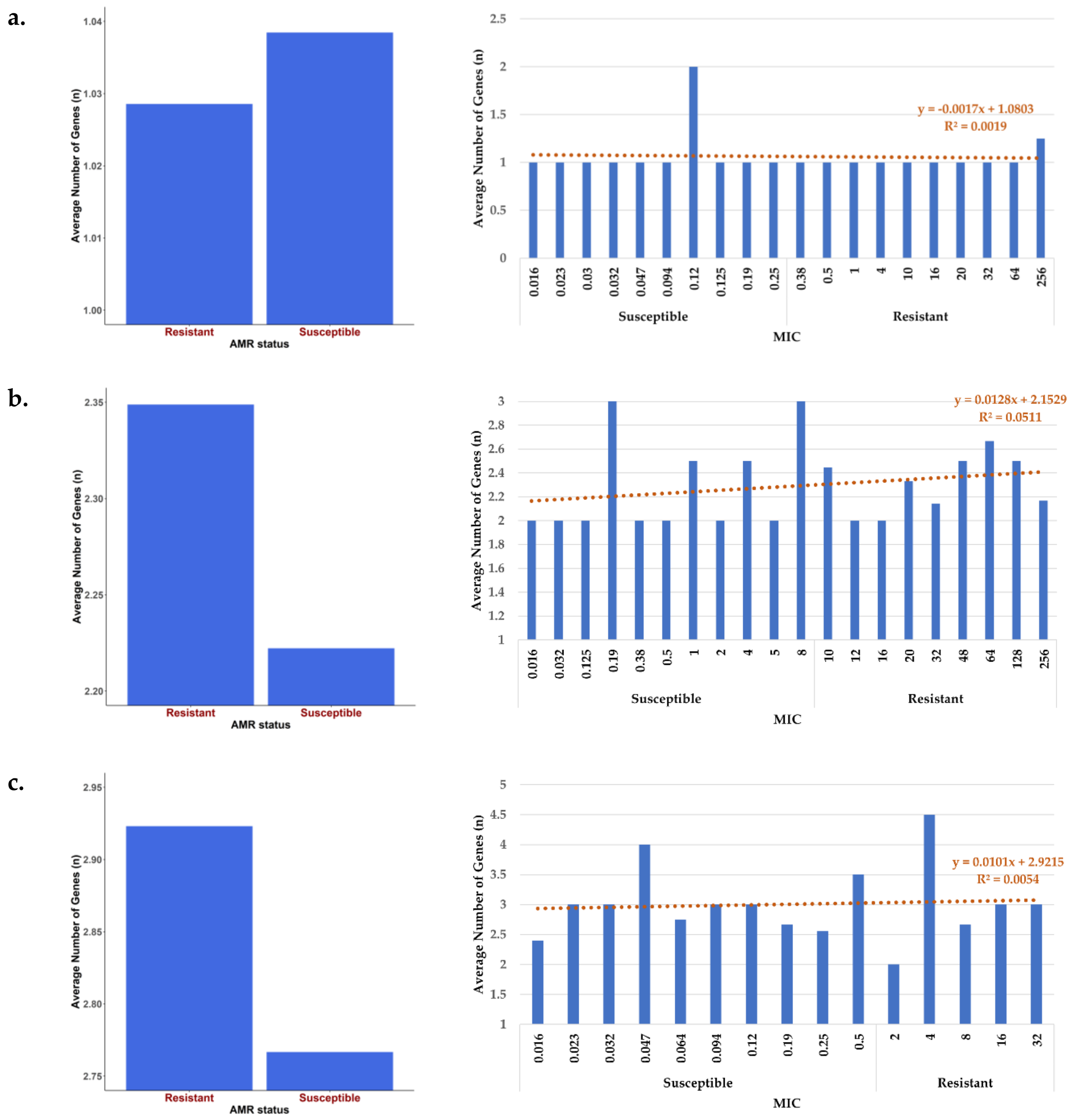

Additional Figure. Comparison and trends of ARG numbers between resistant and susceptible *H. pylori* clinical isolates and their MIC data. Strains with higher MIC tend to have higher number of ARG. **a.** ARG related to clarithromycin resistance. **b.** ARG related to metronidazole resistance. **c.** ARG related to levofloxacin resistance.

Supplementary Table

Table S1. EPs related to AMR reported in *H. pylori*. Most of these genes were already mentioned by recent original and review studies [1,2]. The EPs gene name, length, and GC content were retrieved from *H. pylori* strain 26695 (NC_000915.1).

Locus tag	Gene name (product)	Gene length (bp)	GC content	EPs family	Antibiotic Target	Reference
HP0605	hefA (efflux RND transporter outer membrane subunit HefA)	1434	40.1	RND	Bilesalt, Cefotaxime, Ceragenin, Clindamycine, Clarithromycin, Erythromycin, Ethidium bromide (EtBr), Novobiocin, Metal ion, Nickel, Sodium Deoxycholate, Tetracycline	[3-6]
HP0606	hefB (efflux RND transporter periplasmic adaptor subunit HefB)	1434	40.1	RND	Bilesalt, Cefotaxime, Ceragenin, Clindamycine, Clarithromycin, Erythromycin, EtBr, Novobiocin, Metal ion, Nickel, Sodium Deoxycholate, Tetracycline	[3-6]
HP0607	hefC (efflux RND transporter permease subunit HefC)	3087	42.4	RND	Bilesalt, Cefotaxime, Ceragenin, Clindamycine, Clarithromycin, Erythromycin, EtBr, Novobiocin, Metal ion, Nickel, Sodium Deoxycholate, Tetracycline	[3-6]
HP0759	- (MATE family efflux transporter)	1299	38.8	MATE	unknown	[7]
HP0791	cadA (heavy metal translocating P-type ATPase)	2061	42.6	ABC	Cadmium, zinc	[1]
HP0969	- (CusA/CzcA family heavy metal efflux RND transporter)	3060	41.41	RND	Cadmium, Metronidazole, Nickel, Zinc	[3,5,8]
HP0970	- (efflux RND transporter periplasmic adaptor subunit)	1080	40.09	RND	Cadmium, Metronidazole, Nickel, Zinc	[3,5,8]
HP0971	- (TolC family protein)	1302	38.86	RND	Cadmium, Metronidazole, Nickel, Zinc	[3,5,8]
HP1072	- (glycine--tRNA ligase subunit beta)	2238	39.86	ABC	Copper	[1]
HP1082	- (ABC transporter ATP-binding protein)	1656	38.35	ABC	Erythromycin, EtBr, novobiocin, rifampin, and lipopolysaccharide	[7]

HP1165	- (HP1165 family MFS efflux transporter)	1161	37.9	MFS	Tetracycline	[9]
HP1174	- (sugar MFS transporter)	1224	42.48	MFS	D Glactose (non – drug)	[10]
HP1181	- (MFS transporter)	1332	40.47	MFS	Nitroimidazole, Tetracycline, and Fluoroquinolone	[11]
HP1184	- (HP1184 family multidrug efflux MATE transporter)	1380	39.35	MATE	Norfloxacin and Ethidium	[11]
HP1250	- (SH3 domain-containing protein)	579	40.76	ABC	unknown	[1]
HP1251	- (microcin C ABC transporter permease YejB)	1047	40.78	ABC	unknown	[1]
HP1252	- (extracellular solute-binding protein)	1785	39.78	ABC	unknown	[1]
HP1327	crdB (copper resistance outer membrane protein CrdB)	1239	33.98	RND	Copper, Cobalt, Zinc Cadmium Ion	[4,5]
HP1328	- (efflux RND transporter periplasmic adaptor subunit)	1017	37.36	RND	Copper, Cobalt, Zinc Cadmium Ion	[4,5]
HP1329	- (CusA/CzcA family heavy metal efflux RND transporter)	3108	41.73	RND	Copper, Cobalt, Zinc Cadmium Ion	[4,5]
HP1487	- (ABC transporter permease)	1098	40.53	RND	Novobiocin, Deoxycholate, EtBr resistance	[12]
HP1488	- (RND transporter periplasmic adaptor subunit)	990	43.43	RND	Novobiocin, Deoxycholate, EtBr resistance	[12]
HP1489	- (TolC family protein)	1533	40.38	RND	Novobiocin, Deoxycholate, EtBr resistance	[12]
HP1503	- (heavy metal translocating P-type ATPase)	2367	40.35	ABC	Metal	[1]
HP1561	- (ABC transporter substrate-binding protein)	1008	40.87	ABC	Nickel, Copper	[1,13]

Reference:

1. Raj, D.S.; Kumar Kesavan, D.; Muthusamy, N.; Umamaheswari, S. Efflux pumps potential drug targets to circumvent drug Resistance – Multi drug efflux pumps of Helicobacter pylori. *Materials Today: Proceedings* **2021**, *45*, 2976-2981, doi:10.1016/j.matpr.2020.11.955.
2. Liu, Y.; Wang, S.; Yang, F.; Chi, W.; Ding, L.; Liu, T.; Zhu, F.; Ji, D.; Zhou, J.; Fang, Y.; et al. Antimicrobial resistance patterns and genetic elements associated with the antibiotic resistance of Helicobacter pylori strains from Shanghai. *Gut pathogens* **2022**, *14*, 14, doi:10.1186/s13099-022-00488-y.
3. Waidner, B.; Melchers, K.; Ivanov, I.; Loferer, H.; Bensch, K.W.; Kist, M.; Bereswill, S. Identification by RNA profiling and mutational analysis of the novel copper resistance determinants CrdA (HP1326), CrdB (HP1327), and CzcB (HP1328) in Helicobacter pylori. *J Bacteriol* **2002**, *184*, 6700-6708, doi:10.1128/JB.184.23.6700-6708.2002.
4. Cagliero, C.; Mouline, C.; Cloeckaert, A.; Payot, S. Synergy between efflux pump CmeABC and modifications in ribosomal proteins L4 and L22 in conferring macrolide resistance in Campylobacter jejuni and Campylobacter coli. *Antimicrob Agents Chemother* **2006**, *50*, 3893-3896, doi:10.1128/AAC.00616-06.
5. Bina, J.E.; Alm, R.A.; Uria-Nickelsen, M.; Thomas, S.R.; Trust, T.J.; Hancock, R.E. Helicobacter pylori uptake and efflux: basis for intrinsic susceptibility to antibiotics in vitro. *Antimicrob Agents Chemother* **2000**, *44*, 248-254, doi:10.1128/AAC.44.2.248-254.2000.
6. Hirata, K.; Suzuki, H.; Nishizawa, T.; Tsugawa, H.; Muraoka, H.; Saito, Y.; Matsuzaki, J.; Hibi, T. Contribution of efflux pumps to clarithromycin resistance in Helicobacter pylori. *Journal of gastroenterology and hepatology* **2010**, *25 Suppl 1*, S75-79, doi:10.1111/j.1440-1746.2009.06220.x.
7. Miyamae, S.; Ueda, O.; Yoshimura, F.; Hwang, J.; Tanaka, Y.; Nikaido, H. A MATE family multidrug efflux transporter pumps out fluoroquinolones in Bacteroides thetaiotaomicron. *Antimicrob Agents Chemother* **2001**, *45*, 3341-3346, doi:10.1128/AAC.45.12.3341-3346.2001.
8. Stahler, F.N.; Odenbreit, S.; Haas, R.; Wilrich, J.; Van Vliet, A.H.; Kusters, J.G.; Kist, M.; Bereswill, S. The novel Helicobacter pylori CznABC metal efflux pump is required for cadmium, zinc, and nickel resistance, urease modulation, and gastric colonization. *Infection and immunity* **2006**, *74*, 3845-3852, doi:10.1128/IAI.02025-05.
9. Li, Y.; Dannelly, H.K. Inactivation of the putative tetracycline resistance gene HP1165 in Helicobacter pylori led to loss of inducible tetracycline resistance. *Arch Microbiol* **2006**, *185*, 255-262, doi:10.1007/s00203-006-0093-9.
10. Ge, X.; Cai, Y.; Chen, Z.; Gao, S.; Geng, X.; Li, Y.; Li, Y.; Jia, J.; Sun, Y. Bifunctional Enzyme SpoT Is Involved in Biofilm Formation of Helicobacter pylori with Multidrug Resistance by Upregulating Efflux Pump Hp1174 (gluP). *Antimicrob Agents Chemother* **2018**, *62*, doi:10.1128/AAC.00957-18.
11. Falsafi, T.; Ehsani, A.; Attaran, B.; Niknam, V. Association of hp1181 and hp1184 Genes With the Active Efflux Phenotype in Multidrug-Resistant Isolates of Helicobacter pylori. *Jundishapur J Microbiol* **2016**, *9*, e30726, doi:10.5812/jjm.30726.
12. van Amsterdam, K.; Bart, A.; van der Ende, A. A Helicobacter pylori TolC efflux pump confers resistance to metronidazole. *Antimicrob Agents Chemother* **2005**, *49*, 1477-1482, doi:10.1128/AAC.49.4.1477-1482.2005.
13. Nielsen, D.; Skovsgaard, T. P-glycoprotein as multidrug transporter: a critical review of current multidrug resistant cell lines. *Biochim Biophys Acta* **1992**, *1139*, 169-183, doi:10.1016/0925-4439(92)90131-6.
